# Study on Cooling Layer and Thin Insert Thickness Between Coolant and Cavity for Injection Mold with Bridge-Type Composite Product

**DOI:** 10.3390/polym17212823

**Published:** 2025-10-23

**Authors:** Tran Minh The Uyen, Pham Son Minh, Hung-Son Dang, Bui Chan Thanh

**Affiliations:** 1 Faculty of Mechanical Engineering, HCMC University of Technology and Education, Ho Chi Minh City 71307, Vietnam; uyentmt@hcmute.edu.vn (T.M.T.U.); minhps@hcmute.edu.vn (P.S.M.); 2 Faculty of Vehicle and Energy Engineering, HCMC University of Technology and Education, Ho Chi Minh City 71307, Vietnam; sondh@hcmute.edu.vn

**Keywords:** thin-walled mold, cooling layer, displacement behavior, mold temperature, injection molding, injection pressure, Taguchi method, bridge-type composite

## Abstract

This study focuses on the design and optimization of a cooling layer system integrated into a thin-thickness mold insert to enhance heat transfer efficiency, control mold temperature, and improve the quality of composite products during injection molding. The Taguchi method with an L25 (5^4^) orthogonal array was employed to investigate four key parameters: insert thickness, cooling layer thickness, water flow rate, and coolant temperature. Among 25 experimental combinations, five representative cases were selected for detailed analysis. The results indicate that the optimal configuration (0.5 mm insert, 10 mm cooling layer, 3.5 L/min flow rate, and 80 °C coolant temperature) successfully maintained a high and stable mold temperature, with a cavity temperature difference of only 3.6 °C at steady state and a simulation–experiment deviation ranging from 2.4% to 7.2%. This condition not only improved melt flowability and surface quality but also reduced defects such as weld lines, warpage, and shrinkage. In parallel, displacement measurements on PA6 and glass fiber-reinforced PA6 (PA6 + GF) composites revealed that increasing the fiber content from 0% to 30% reduced output displacement by more than 19% compared to neat PA6, highlighting the reinforcing effect of glass fibers and the relationship between temperature distribution and mechanical displacement behavior. The findings confirm that integrating a cooling layer into a thin-thickness mold, combined with Taguchi-based optimization, provides an effective approach to enhance through-thickness heat transfer, reduce deformation, and ensure the overall quality of composite injection-molded products in industrial applications.

## 1. Introduction

In modern plastics manufacturing, thin-walled injection molding has emerged as a dominant trend owing to its capacity to reduce product weight, optimize material efficiency, shorten production cycles, and enhance geometric accuracy [1,2]. This technology is pivotal in demanding sectors such as automotive, consumer electronics, biomedical devices, and precision engineering [3,4]. However, producing parts with wall thicknesses below 1 mm, intricate geometries, and stringent surface requirements presents significant challenges for the injection molding process, particularly in mold temperature control, thermal uniformity, and deformation mitigation [5]. As wall thickness decreases, the reduced heat transfer area results in a steep through-thickness thermal gradient. This in turn leads to non-uniform shrinkage, geometric warpage, sink marks, and diminished dimensional stability [6,7]. To mitigate these issues, recent research has predominantly focused on two approaches: designing thin-walled mold inserts and integrating advanced cooling systems. Among these, the Cooling Layer concept has emerged as a particularly promising solution [8].

The design of thin-walled mold inserts is pivotal for enhancing through-thickness heat transfer and reducing cooling time. As the insert thickness decreases, the thermal resistance of the conduction path is reduced, thereby promoting a more uniform temperature distribution and optimizing thermal efficiency [9]. Experimental studies have demonstrated that reducing insert thickness from 12 mm to 4 mm can increase the through-thickness heat transfer rate by up to 35% and shorten the average cooling time by approximately 25–30% [10]. However, thinner inserts also render the mold system more susceptible to temperature fluctuations. This can lead to non-uniform thermal distribution, higher residual stresses, and product warpage that is difficult to control without effective thermal management [11]. This issue is exacerbated in products with intricate geometries and stringent requirements for dimensional accuracy or surface quality [12].

To mitigate this thermal management challenge, several technologies have been developed. While resistance heating can accelerate heating, achieving uniform surface temperature is difficult [13]. Induction heating provides faster heating rates but is more energy-intensive and offers limited compatibility with thin-walled inserts [14]. Conformal cooling channels improve localized cooling, but their effectiveness diminishes with decreasing insert thickness [15]. In contrast, the Cooling Layer system emerges as a superior solution. By positioning a specialized thermally conductive layer directly beneath the insert surface, this system simultaneously enhances through-thickness heat transfer, reduces the temperature differential (ΔT), and promotes a highly uniform surface temperature, ultimately leading to superior product quality [16,17].

The efficacy of the Cooling Layer has been well-documented in numerous experimental and simulation studies. Studies have reported that the Cooling Layer can reduce cooling time by 40–60% compared to conventional channels. Concurrently, it decreases the mold surface temperature differential (ΔT) from 18 °C to 6 °C, thereby stabilizing thermal conditions throughout the injection molding cycle [18,19]. This enhanced thermal control serves to minimize sink marks, improve surface quality, and reduce product warpage by up to 35% [20]. Thermodynamic analyses further reveal that the Cooling Layer increases average through-thickness heat transfer efficiency by 25% and significantly improves temperature uniformity, particularly in mold regions with intricate geometries [21]. Moreover, the Cooling Layer facilitates an approximate 30% reduction in overall residual stress, thereby mitigating nonlinear deformation and improving the dimensional accuracy of composite products [22]. Select studies have also reported that by maintaining uniform cooling, the Cooling Layer can optimize glass fiber orientation in composites such as PA6 with 30 wt.% glass fiber (PA6/30 wt.% GF). This in turn reduces displacement behavior and enhances the mechanical homogeneity of molded parts [23].

Numerous studies have employed methods such as the Taguchi design, Response Surface Methodology (RSM), and numerical simulations to optimize Cooling Layer configurations. These optimization efforts have demonstrated that adjusting the location, number, and dimensions of the layers can reduce the mold surface temperature differential (ΔT) by up to 70%, shorten the average cooling time by 45%, and decrease overall deformation by 30–40%, while simultaneously improving the dimensional accuracy of composite products [24,25].

Beyond technological solutions, the choice of engineering polymer is also critical to the quality of thin-walled parts. Among these materials, polyamide 6 (PA6) and its composite reinforced with 30 wt.% glass fiber (PA6/30 wt.% GF) are frequently utilized for their low density, high mechanical strength, and good thermal resistance [26]. However, PA6 GF composites exhibit pronounced thermo-mechanical anisotropy, rendering them highly susceptible to thermal gradients, which often results in non-uniform shrinkage, complex warpage, and nonlinear displacement behavior [27]. Experimental results have revealed that increasing the glass fiber content from 0% to 30% correlates with an approximate 30% increase in elastic modulus; however, the material’s sensitivity to thermal variations also increases, exacerbating product deformation [28]. Notably, integrating Cooling Layers into the injection molding of PA6/30 wt.% GF composites has been shown to yield substantial benefits. These include the stabilization of fiber orientation, an approximate 25% reduction in residual stress, an overall warpage reduction of 35%, and a significant enhancement of surface quality [29,30].

Research on compliant mechanisms has garnered significant interest for applications in precision micro-positioning, displacement sensing, and metrology. Numerous studies have focused on design and optimization approaches to amplify displacement and enhance positioning accuracy. For instance, a study by [31] presented a one-degree-of-freedom (1-DOF) compliant mechanism fabricated via additive manufacturing. Their results demonstrated that the bridge-type design improved displacement accuracy by up to 18% compared to conventional mechanisms. Building on this research, ref. [32] proposed a two-degree-of-freedom (2-DOF) mechanism that synergized kinematic–static analysis with artificial neural network (ANN) modeling. This hybrid approach reduced deformation prediction errors to below 5% and enhanced micro-positioning capabilities. More recently, ref. [33] designed an XYZ micropositioner with an integrated displacement sensor, achieving a positioning accuracy of ±2 µm. This level of precision, demonstrated in metrology and biological sample probing, highlights its potential for advanced measurement systems.

While advanced cooling solutions such as conformal cooling [34,35] and microchannel cooling [16] have demonstrated efficacy in enhancing heat transfer, reducing cycle times, and mitigating warpage in injection molding, they exhibit significant limitations, particularly for thin-walled molds (less 1 mm thickness). Specifically, conformal cooling channels impose significant spatial requirements and can compromise the mechanical integrity of the mold insert. Meanwhile, while microchannels offer high heat exchange efficiency, they pose considerable fabrication challenges, are susceptible to clogging, and fail to ensure thermal stability under mass production conditions [34]. Furthermore, the majority of existing research has predominantly focused on thermal efficiency or cooling cycle optimization. A significant research gap persists in the analysis of thermo-mechanical interactions needed to comprehensively evaluate the displacement behavior and non-linear deformation of composite materials during injection molding [36].

In contrast to the aforementioned methods, this study proposes a novel thin-walled mold design that integrates a Cooling Layer system directly beneath the insert. This design utilizes a specialized heat-conducting layer to shorten the through-thickness heat transfer path, reduce the surface temperature differential (ΔT), and enhance thermal uniformity throughout the injection cycle [36,37,38]. A key innovation of this work is the pioneering integration of Taguchi-based parameter optimization with coupled thermo-mechanical simulations and experimental deformation measurements. This approach enables an in-depth analysis of the heat transfer–deformation mechanisms of PA6/PA6-GF materials under thin-wall injection molding conditions [39]. This holistic approach not only addresses the limitations of conventional conformal and microchannel cooling but also establishes a new design framework. This framework is capable of enhancing heat transfer efficiency, reducing deformation, controlling residual stress, and significantly improving the quality of composite products in engineering applications that demand high geometrical precision [40].

While previous studies have established that integrating thin-walled mold inserts with Cooling Layers yields significant advantages, these benefits have often been investigated in isolation. Such advantages include enhanced heat transfer efficiency, improved thermal uniformity, mitigated residual stress and warpage, and optimized fiber orientation. However, most existing works have focused on discrete aspects, such as thin-walled insert design [9,10], Cooling Layer optimization [18,24], or the warpage analysis of PA6 GF composites [27,29]. To date, a comprehensive study that simultaneously combines thin-walled insert design, Cooling Layer integration, and the displacement behavior analysis of PA6/GF composites via a coupled experimental and thermo-mechanical simulation approach is notably absent from the literature. This research gap provides the primary rationale for the present study. We aim to design, optimize, and experimentally validate an integrated Cooling Layer system within thin-walled inserts, while concurrently analyzing the displacement behavior and overall deformation of PA6/GF composite products. The ultimate goal is to propose an optimized mold design framework that simultaneously enhances heat transfer efficiency, minimizes warpage and shrinkage, and improves the overall quality of thin-walled composite products.

Building upon the identified research gap, this work aims not only to confirm the benefits of thin-walled inserts and the Cooling Layer system but also to formulate and test verifiable scientific hypotheses. The objective is to elucidate the thermo-mechanical interaction mechanisms during composite injection molding. Specifically, it is hypothesized that reducing the insert thickness in conjunction with increasing the thickness and thermal conductivity of the Cooling Layer will significantly mitigate the through-thickness thermal gradient (ΔT). This, in turn, is expected to improve mold surface temperature distribution, reduce residual stress, and consequently decrease the non-linear deformation and overall displacement of PA6/GF composite products [37,40,41]. Furthermore, optimizing the Cooling Layer configuration is anticipated to increase through-thickness heat transfer efficiency by over 25% compared to traditional cooling systems and reduce the surface ΔT from 18 °C to below 6 °C. These improvements are projected to enhance the surface quality and geometrical accuracy of the final product [13,36,37].

## 2. Materials and Methods

### 2.1. Geometry and Preparation of Composite Sample

**Figure 1:**
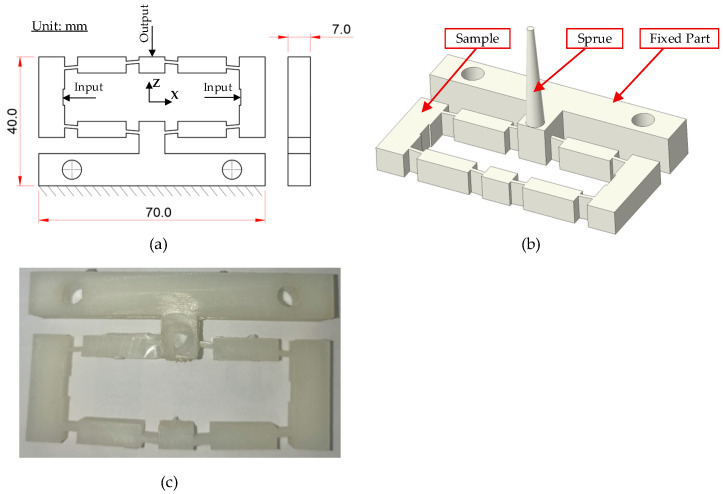
Geometry and dimensions of the composite displacement measurement sample: (**a**) 2D schematic illustrating dimensional parameters and the defined coordinate system; (**b**) 3D model representation highlighting the primary functional regions: sample, sprue, and fixed part; (**c**) Photograph of the injection-molded specimen.

The test specimen features overall dimensions of 70 mm × 40 mm × 7 mm and is designed with three main sections: (i) a sample region, representing the product zone subjected to thermo-mechanical effects; (ii) a fixed part, which ensures structural stability during displacement measurements; and (iii) a gate, through which the molten polymer enters the cavity. A detailed 2D drawing is presented in Figure 1a, specifying dimensional parameters, displacement directions (X–Y), and defined input–output positions for subsequent deformation analysis. The 3D model, depicted in Figure 1b, shows the distinctly separated functional regions, a design that facilitates both integration into the mold system and use in flow and heat transfer simulations.

Figure 1c illustrates the actual molded specimen, fabricated from polyamide 6 (PA6) and PA6 reinforced with 30 wt.% glass fiber (PA6/30 wt.% GF). The material selection was predicated on its superior thermo-mechanical properties—including high tensile strength, stiffness, and thermal resistance—coupled with its known sensitivity to thermal gradients, which can induce complex geometric deformations. The specimen’s geometry was intentionally designed to induce regions of varying stress concentration. This enables a comprehensive evaluation of its displacement behavior under the diverse operating conditions of the thin-walled insert and Cooling Layer system.

### 2.2. Taguchi Design and Experimental Parameters

In this study, the Taguchi method was employed to systematically investigate and optimize the key process parameters affecting mold temperature control and the displacement characteristics of composite products. This method utilizes an Orthogonal Array (OA) to significantly reduce the number of experimental runs compared to a full factorial design (which would require 5^4^ = 625 experiments). Crucially, the OA approach maintains statistical validity and allows for the independent evaluation of each factor’s contribution [42,43,44]. Accordingly, four key factors were selected for investigation—insert thickness, cooling layer thickness, coolant flow rate, and coolant temperature—each at five levels (Table 1). An L_25_ (5^4^) orthogonal array was utilized to structure the 25 experimental runs, facilitating the analysis of both main effects and potential interactions within the cooling system of the thin-walled mold insert.

To optimize the experimental workflow while ensuring comprehensive coverage of the design space, five representative cases were selected from the L_25_ Taguchi matrix (Table 2). These cases, numbered 1, 5, 8, 17, and 22, were chosen to capture both extreme and intermediate conditions:

- Case 1: 0.5 mm insert thickness, 2 mm cooling layer thickness, 1.5 L/min flow rate, 40 °C water temperature.
- Case 5: 0.5 mm insert thickness, 10 mm cooling layer thickness, 3.5 L/min flow rate, 80 °C water temperature.
- Case 8: 1.0 mm insert thickness, 8 mm cooling layer thickness, 2.5 L/min flow rate, 80 °C water temperature.
- Case 17: 2.0 mm insert thickness, 10 mm cooling layer thickness, 2.0 L/min flow rate, 60 °C water temperature.
- Case 22: 2.5 mm insert thickness, 2 mm cooling layer thickness, 2.0 L/min flow rate, 80 °C water temperature.

This selection encompasses a range of conditions, from aggressive to mild cooling, and includes diverse geometric features of the insert. Consequently, it provides a robust dataset for the comparative analysis and validation of the numerical model. This resource-efficient approach provides a solid foundation for identifying the most influential factors and evaluating the relationships between mold geometry, the Cooling Layer system, and process parameters in injection molding.

### 2.3. Mold Design and Cooling Layer Configuration

**Figure 2:**
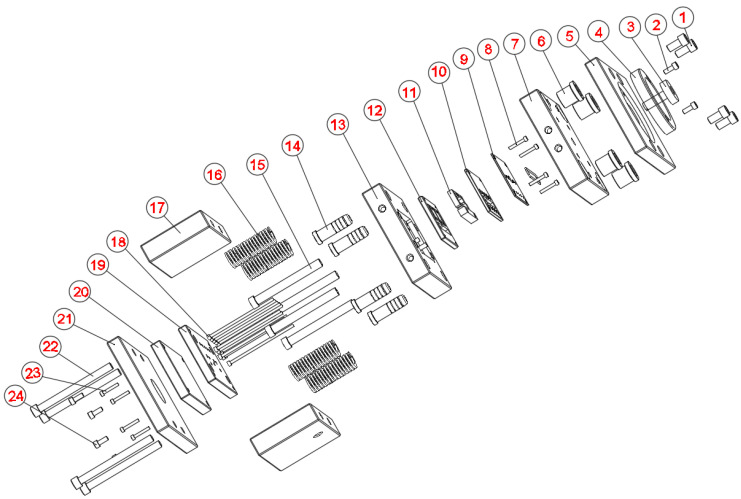
Exploded view of the injection mold assembly with cooling layer components.

The injection mold features a thin-walled structure integrated with a Cooling Layer system, a design intended to promote a uniform mold surface temperature and enhance the quality of composite products. Figure 2 depicts the exploded schematic of the injection mold assembly, highlighting key components such as the cavity mold plate, insert plate, sample insert plate, and rubber seal. This configuration creates an independent thermal conduction layer positioned directly beneath the mold surface. This design feature enhances through-thickness heat transfer efficiency compared to conventional cooling channels. Notably, the insert system was designed to be modular, allowing the cooling layer thickness to be varied. This facilitated an investigation into how different configuration parameters influence cooling performance.

**Figure 3:**
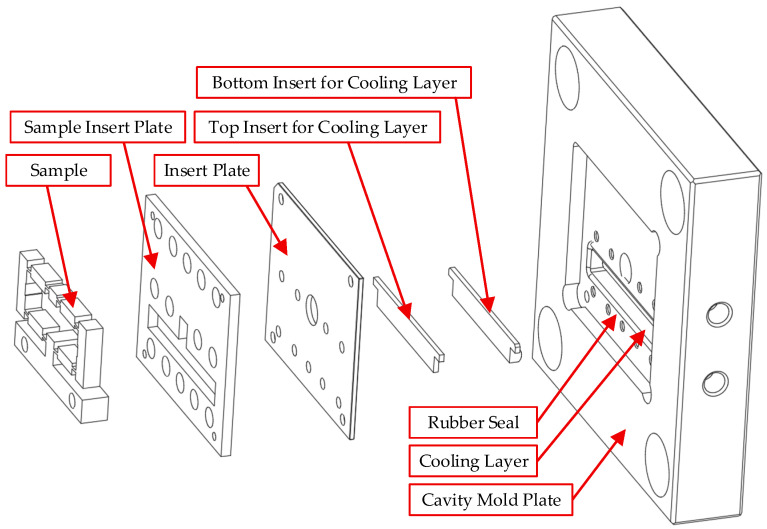
Exploded view of the cavity mold assembly showing the cavity mold plate, cooling layer, rubber seal, insert plate, and sample insert plate.

**Figure 4:**
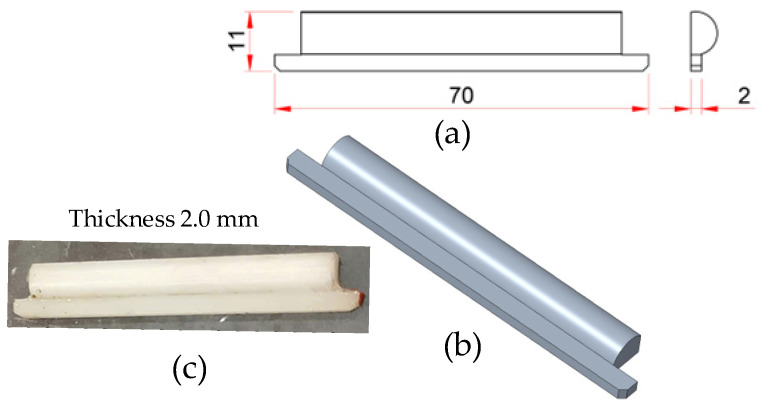
Bottom insert used to form the lower cooling layer in the mold assembly: (**a**) 2D technical drawing illustrating the main dimensions of the bottom insert; (**b**) 3D model representation of the bottom insert; (**c**) Photograph of the fabricated bottom insert.

**Figure 5:**
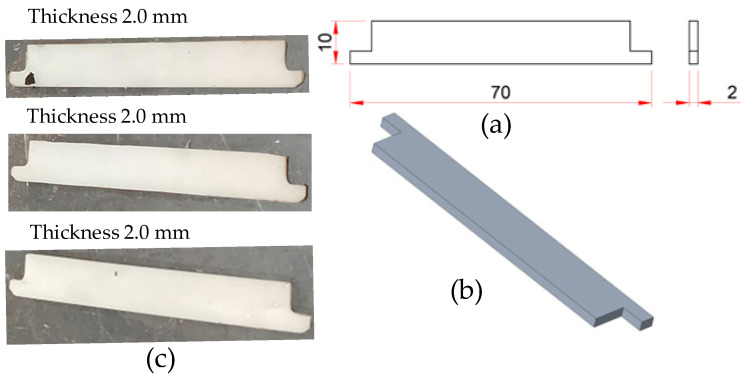
Top insert used to form the lower cooling layer in the mold assembly: (**a**) 2D technical drawing illustrating the main dimensions of the top insert; (**b**) 3D model representation of the top insert; (**c**) Photographs of the fabricated top inserts.

Figure 3 details the cavity mold assembly, which comprises the cavity mold plate, cooling layer, rubber seal, insert plate, and sample insert plate. These components are integrated into a unified block to ensure a robust seal for coolant circulation within the Cooling Layer. The base of the cooling chamber is formed by a 2 mm-thick bottom plate, which also acts as a flow barrier (Figure 4). This configuration defines a nominal water layer thickness of 10 mm. Conversely, the upper insert system (Figure 5) is a modular assembly of three 2 mm-thick plates. This design allows the overall thickness of the cooling layer to be varied from 2 mm to 10 mm.

This configuration forms a planar coolant channel that enhances heat transfer efficiency relative to conventional drilled channels. Crucially, this versatility enables the system to accommodate all experimental cases defined by the Taguchi design. All inserts were manufactured with high dimensional accuracy to ensure precise assembly and promote optimal coolant flow behavior during operation.

The combination of the top and bottom inserts creates a defined circulation gap for the coolant, directly governing both the heat transfer rate and the thermal gradient across the thin-walled mold. Rubber seals were strategically positioned to prevent leakage and maintain the integrity of the Cooling Layer during cyclic thermal processes. This modular design offers significant flexibility, not only enabling the adjustment of cooling parameters but also facilitating ease of assembly, replacement, and maintenance.

**Figure 6:**
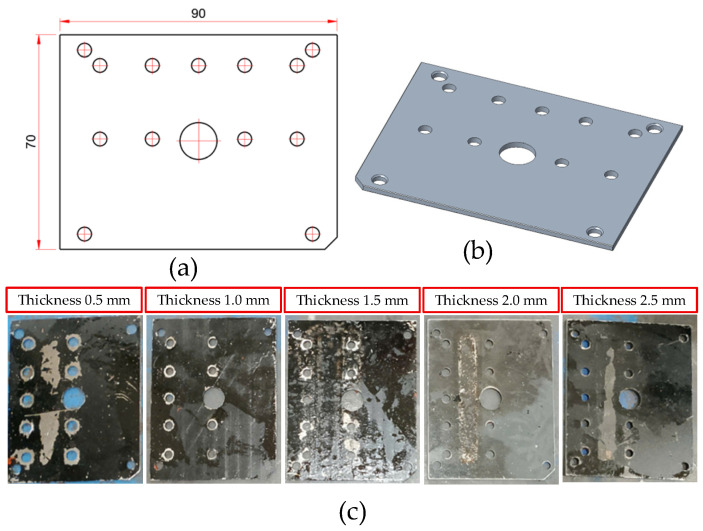
Insert Plate used to form the lower cooling layer in the mold assembly: (**a**) 2D technical drawing illustrating the dimensional specifications of the insert plate; (**b**) 3D model representation of the insert plate; (**c**) Photographs of the fabricated insert plates with varying thicknesses (0.5 mm to 2.5 mm).

As depicted in Figure 6, the thin insert plate is the most critical component of the Cooling Layer system. Functioning as the primary thermal interface between the product cavity and the coolant, all heat dissipated from the composite product must pass through it. Consequently, the insert thickness is a highly influential parameter, directly affecting the cooling rate, surface temperature uniformity, residual stress development, and product warpage. To comprehensively evaluate these effects, insert plates were fabricated at five thickness levels (0.5, 1.0, 1.5, 2.0, and 2.5 mm). This range of thicknesses enables an investigation into the trade-off between cooling efficiency and the mechanical stiffness of the mold, facilitating the identification of an optimal threshold that ensures both effective heat transfer and structural rigidity.

**Figure 7:**
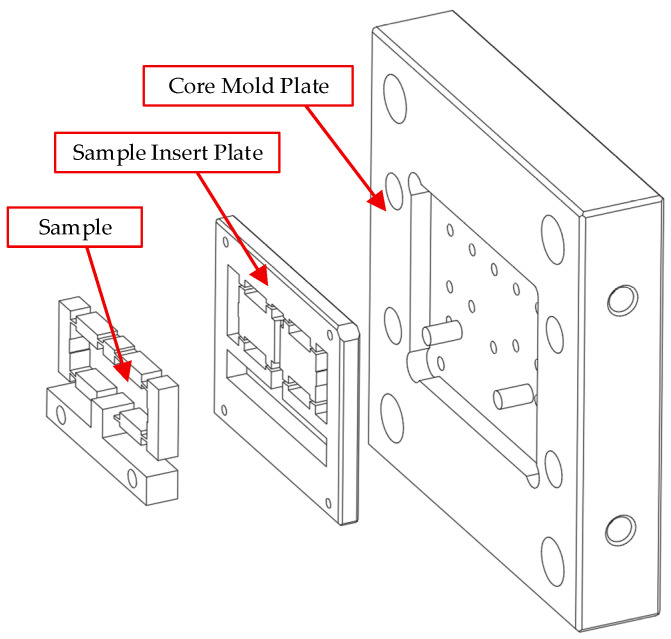
Exploded view of the core mold assembly showing the core mold plate, sample insert plate, and composite sample.

Figure 7 depicts the exploded view of the core mold assembly, which comprises two main components: the core mold plate and the sample insert plate. These components collectively define the cavity where the composite sample is formed. The core mold plate provides the structural foundation, ensuring overall rigidity and system stability against the pressures exerted during the injection process. Directly mounted onto this plate, the sample insert plate serves a dual function: it precisely defines the product geometry while also acting as the primary medium for heat transfer from the molded part to the cooling system. Its role is therefore critical, as it directly governs both the surface quality and dimensional accuracy of the final product.

The mold cavity, formed at the interface between the sample insert plate and the core mold plate, is specifically designed to facilitate the direct investigation of the displacement behavior of PA6 and PA6 reinforced with different glass fiber contents. This provides valuable insight into the thermo-mechanical performance of fiber-reinforced composites in thin-walled injection molding.

### 2.4. Experimental Setup and Procedure

#### 2.4.1. Injection Mold Experimental Setup

**Figure 8:**
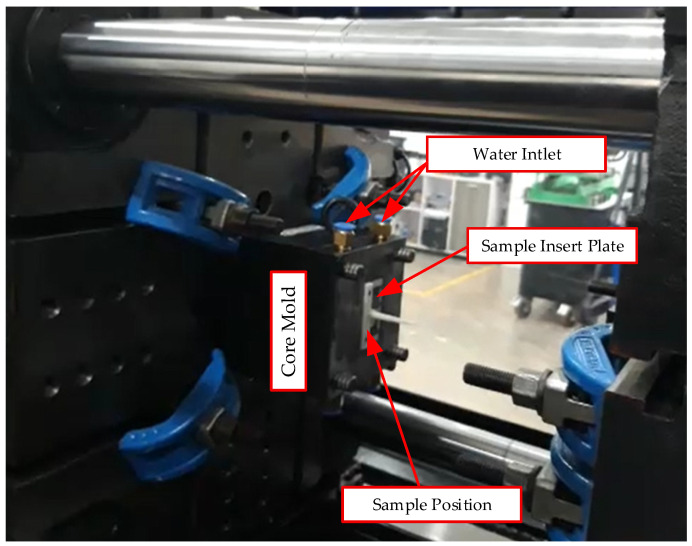
Core mold assembly mounted on the injection molding machine.

Figure 8 depicts the core mold assembly mounted on the injection molding machine, highlighting key components such as the core mold plate, the sample insert plate, and the water inlet system. The injection molding conditions for this study corresponded to Case 5 of the Taguchi matrix. The parameters for this case were defined as follows: a 0.5 mm insert thickness, a 10 mm cooling layer thickness, a 3.5 L/min coolant flow rate, and an 80 °C water temperature. This configuration was selected to facilitate stable high-temperature control and optimize through-thickness heat transfer. These conditions are intended to promote uniform cavity filling of the PA6/30 wt.% GF composite melt while concurrently mitigating shrinkage and geometric deformation.

To investigate the influence of glass fiber content on displacement behavior, PA6/GF composites were prepared with fiber content systematically varied from 0 wt.% (neat PA6) to 30 wt.% in 5 wt.% increments (Table 3). This allowed for a comprehensive evaluation of how the reinforcement level affects the thermo-mechanical response. Neat PA6 (0 wt.%) served as the baseline, while PA6/30 wt.% GF represented the maximum reinforcement level. With the exception of material composition, all injection molding parameters were held constant across the trials as specified in Table 4, including a filling time of 2 s, a cooling time of 24 s, and a melt temperature of 205 °C. This controlled approach enables an accurate analysis of the Cooling Layer’s effects.

The injection molding trials were conducted on a Haitian MA1200 III all-electric injection molding machine (Haitian Plastics Machinery Group Co., Ltd., Ningbo, China), featuring a maximum clamping force of 1200 kN and an injection pressure capacity of 180 MPa. The machine’s hydraulic injection pressure was set to 1000 bar with a packing pressure of 700 bar. It is important to clarify that these are machine settings, whereas the pressure values reported in Table 4 (e.g., 39 bar filling pressure) represent the corresponding melt pressures within the cavity as derived from the Moldex3D (version 2024) simulation. This distinction is critical, as the simulation values are used for calibrating the numerical model against experimental outcomes.

#### 2.4.2. Experimental Equipment

**Figure 9:**
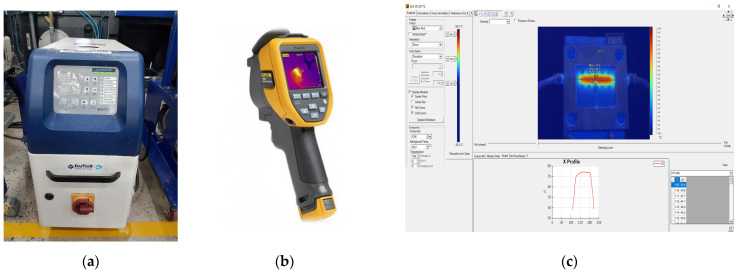
Experimental equipment used in the mold heating and thermal measurement process. (**a**) Mold Heating Unit (Haitian Plastics Machinery Group Co., Ltd., Ningbo, China) [45]. (**b**) Fluke Ti20 Infrared Thermal Camera [46]. (**c**) SmartView 4.4 software used for thermal image analysis and post-processing [47].

Figure 9 illustrates the mold surface temperature measurement system was composed of three integrated instruments to ensure both the accuracy and reliability of the collected data. A Haitian mold heater was employed to maintain stable thermal boundary conditions [45]. The temperature distribution on the mold surface was then captured via a Fluke Ti20 infrared (Fluke Corporation, Everett, WA, USA) thermal camera, a device that affords non-contact measurements with high sensitivity and real-time imaging capabilities, facilitating a precise assessment of the mold’s heat transfer behavior [46]. Finally, the raw thermal data were processed, calibrated, and analyzed using SmartView 4.4 software to generate detailed thermal maps. These maps enabled a comprehensive comparison between experimental measurements and numerical simulation results [47]. This integrated approach constitutes an effective thermal measurement framework, ensuring that the experimental data accurately reflect the thermal state of the mold.

#### 2.4.3. Displacement Measurement Model

**Figure 10:**
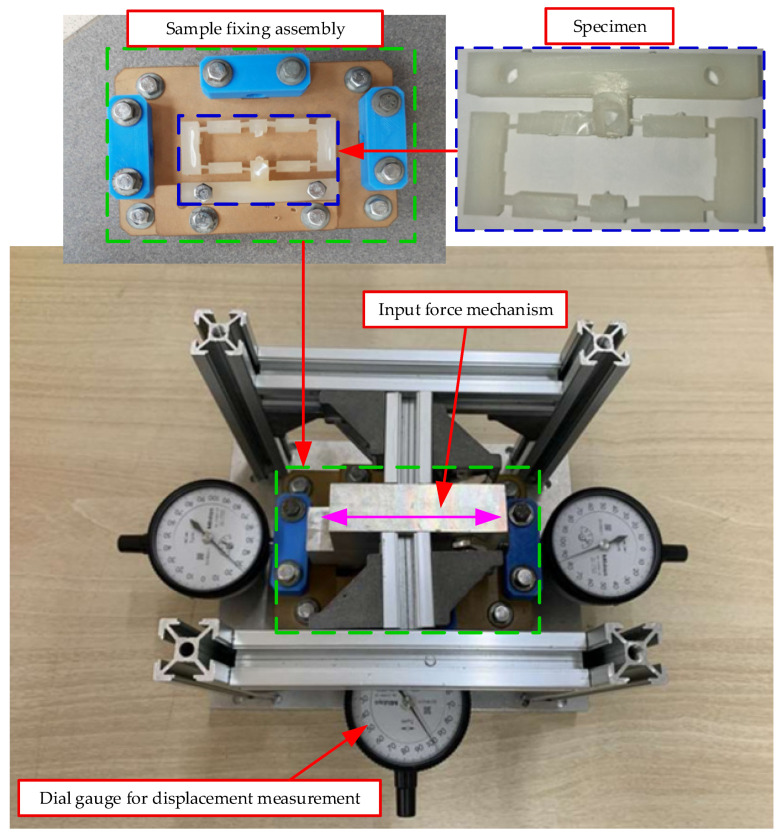
Experimental setup for displacement measurement, including sample fixing assembly, input force mechanism, and dial gauges for displacement recording.

Figure 10 depicts the experimental setup configured to evaluate the deformation characteristics of the injection-molded composite specimens. The apparatus comprises three main components:

(i). A sample fixing assembly to firmly clamp the specimen and prevent undesired movement during testing;
(ii). An input force mechanism to apply a controlled displacement to the input ends of the compliant structure;
(iii). Dial gauges to capture the output displacement response with high accuracy.

In operation, the input force mechanism actuates the specimen—representing the injection-molded PA6 and PA6/GF composite samples—along the X-axis, while the dial gauges simultaneously record the corresponding output displacement along the Z-axis. This experimental configuration enables the assessment of the displacement amplification ratio, mechanical response, and overall deformation behavior of the composites. Furthermore, the precise alignment of all components guarantees the repeatability and accuracy of the results, establishing a robust basis for validation against numerical simulations.

Displacement measurement tests adhered to ASTM D638-14 [48] and ISO 527-1:2019 standards [49] and were conducted in a controlled ambient environment of 23 ± 2 °C and 50 ± 5% relative humidity. A constant loading rate of 1.0 mm/min was applied via an Instron 3369 universal testing machine. During testing, one end of the specimen was rigidly fixed while the other was subjected to axial loading. Displacement was recorded using a linear variable differential transformer (LVDT) sensor featuring a resolution of ±1 µm. These conditions were chosen to ensure elastic deformation under quasi-static loading, thereby providing accurate displacement data for validation against simulation results.

## 3. Results and Discussion

### 3.1. Thermal Analysis

#### 3.1.1. Case 1: Insert Thickness: 0.5 mm—Cooling Layer Thickness: 2 mm—Water Flow Rate: 1.5 L/min—Coolant Temperature: 40 °C

**Figure 11:**
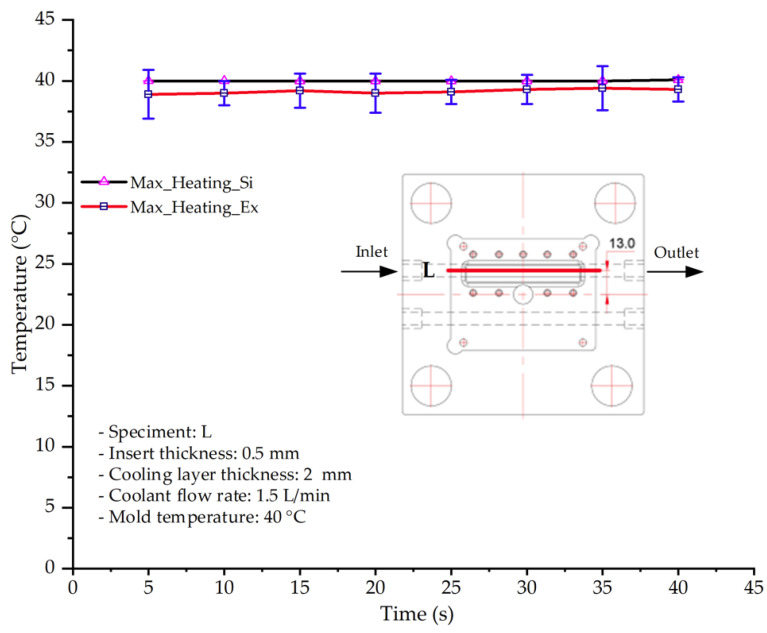
Comparison of maximum heating temperature between simulation and experiment for Case 1.

As shown in Figure 11 and Table 5 and Table 6, the temperature distribution within the mold cavity demonstrates a highly consistent trend between the simulation and experimental methods, despite minor deviations. The simulated maximum temperature ranged from 39.98–40.08 °C, whereas the experimental values were slightly lower, between 38.9–39.4 °C. This deviation, ranging from 1.50% to 2.80%, underscores the strong predictive fidelity of the simulation for maximum temperature.

For the minimum temperature, more pronounced discrepancies were observed. Simulation values ranged from 34.84–38.52 °C, while the experimental data were confined to a narrower range of 37.6–38.6 °C. The resulting percentage error (0.47% to 9.27%) suggests the influence of uncontrolled experimental factors, such as ambient heat exchange and measurement uncertainties. The time-dependent curves from both methods confirm a rapid initial temperature increase (5–15 s) followed by stabilization within 20–40 s. This behavior highlights the effectiveness of the Cooling Layer in maintaining thermal uniformity and mitigating large thermal gradients.

Moreover, the observed discrepancies can be attributed to the divergence between the idealized boundary conditions of the simulation (e.g., uniform surfaces, steady-state flow) and the complexities of the actual experiment, which include turbulence, material heterogeneity, and imperfect thermal contact.

#### 3.1.2. Case 5: Insert Thickness: 0.5 mm—Cooling Layer Thickness: 10 mm—Water Flow Rate: 3.5 L/min—Coolant Temperature: 80 °C

**Figure 12:**
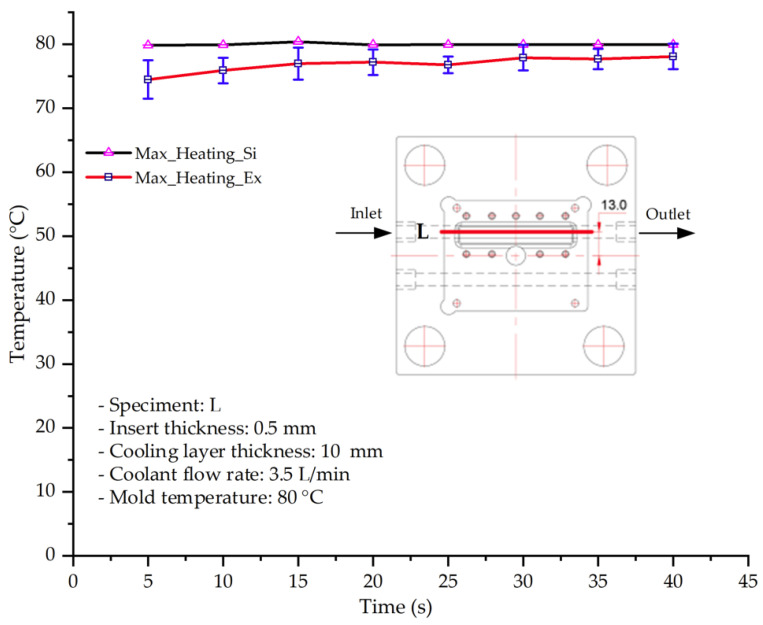
Comparison of maximum heating temperature between simulation and experiment for Case 5.

Under the conditions of Case 5, the temperature distribution showed a strong temporal correlation between simulation and experimental results (Figure 12, Table 7 and Table 8). For the maximum temperature, the simulation recorded a range of 79.86–80.4 °C, whereas experimental values were slightly lower, between 74.5–78.1 °C. The resulting percentage error (2.36% to 7.19%) was higher than that observed at a lower coolant temperature (40 °C). This suggests that at elevated temperatures, the influence of unmodeled experimental factors—such as ambient heat exchange and imperfect surface contact—becomes more pronounced.

For the minimum temperature, simulation values ranged from 67.6–77.76 °C, while experimental results were within 71.0–75.3 °C. The percentage error for this metric (1.52% to 5.85%) reflects persistent deviations between the idealized simulation and actual heat dissipation. Both methods consistently showed a rapid initial temperature increase (5–15 s) followed by a transition to a quasi-steady state after 20–40 s. This underscores the model’s reliability in predicting the overall heat transfer kinetics.

A notable finding is that the 10 mm cooling layer, combined with a high coolant flow rate, promoted a more uniform temperature distribution and effectively mitigated large thermal gradients. However, the remaining deviations suggest that future modeling efforts could be improved by incorporating more realistic boundary conditions. These include surface roughness, complex turbulent flow behavior, and the heterogeneous thermal conductivity of the material.

#### 3.1.3. Case 8: Insert Thickness: 1 mm—Cooling Layer Thickness: 8 mm—Water Flow Rate: 2.5 L/min—Coolant Temperature: 80 °C

**Figure 13:**
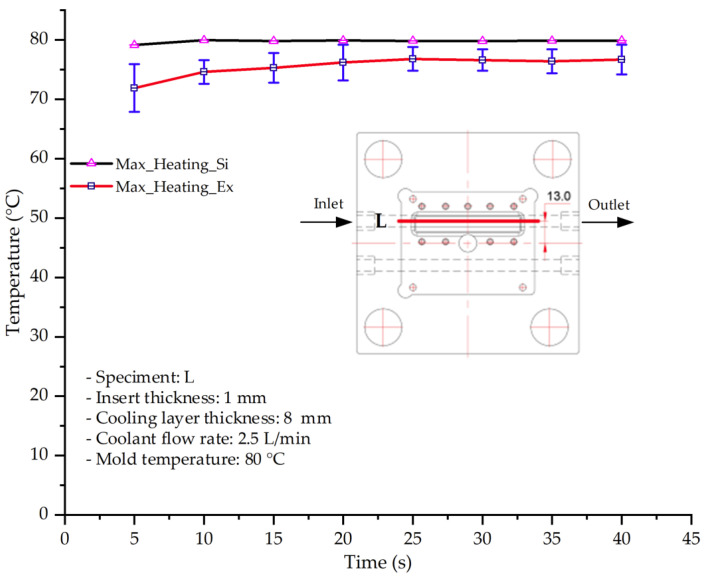
Comparison of maximum heating temperature between simulation and experiment for Case 8.

Under the conditions of Case 8, the temperature distribution showed a general correlation between simulation and experimental results, although significant deviations persisted at certain time points (Figure 13, Table 9 and Table 10). Specifically, the simulated maximum temperature ranged from 79.13–79.94 °C, while the experimental results were lower, ranging from 71.9–76.8 °C. The percentage error for the maximum temperature (3.93% to 10.06%) was higher than in previous cases, suggesting that at elevated temperatures, the influence of unmodeled factors such as heat loss and material heterogeneity becomes more pronounced.

For the minimum temperature, the simulation yielded a range of 66.88–76.95 °C, while experimental measurements were between 66.0–74.1 °C. The corresponding percentage error (0.42% to 5.27%), while lower than that for the maximum temperature, still reflects the divergence between the actual heat dissipation and the model’s idealized assumptions. Notably, a rapid initial temperature rise (5–15 s) followed by stabilization (20–40 s) was observed in both methods, underscoring the model’s ability to capture the overall thermal evolution.

The results also demonstrate that increasing the insert thickness to 1.0 mm leads to a less uniform temperature distribution compared to cases with thinner inserts. This is attributable to the reduced efficiency of heat conduction through the thicker insert, which in turn leads to larger thermal gradients and magnifies the error between simulation and experiment. Therefore, these findings suggest that employing thinner inserts (less 0.5 mm) in conjunction with an optimally designed cooling layer yields superior heat transfer performance and narrows the gap between simulation and experimental outcomes.

#### 3.1.4. Case 17: Insert Thickness: 2 mm—Cooling Layer Thickness: 10 mm—Water Flow Rate: 2.5 L/min—Coolant Temperature: 60 °C

**Figure 14:**
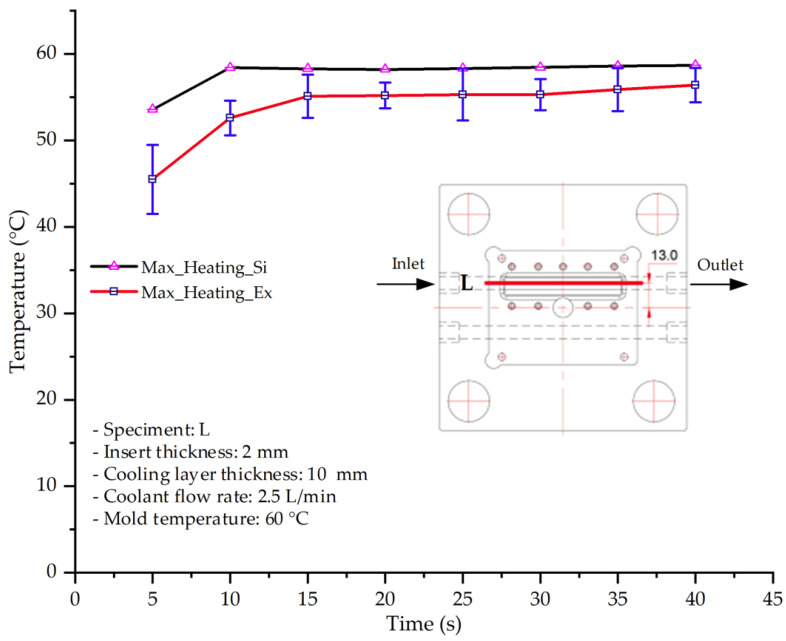
Comparison of maximum heating temperature between simulation and experiment for Case 17.

Under the conditions of Case 17, a general correlation was observed between the simulated and experimental temperature distributions, though with more notable deviations than in previous cases (Figure 14, Table 11 and Table 12). The simulated maximum temperature ranged from 53.57–58.69 °C, whereas the experimental values were lower, ranging from 45.5–56.4 °C. The resulting percentage error for the maximum temperature (4.06% to 17.74%) was significantly higher than in prior cases. This suggests that with a thicker 2.0 mm insert, the increased thermal resistance impedes heat transfer, thereby reducing the simulation’s predictive accuracy.

For the minimum temperature, the simulation yielded a range of 45.06–55.83 °C, while experimental values were between 43–53 °C. The relative error for this metric (4.79% to 12.42%) was also generally higher compared to cases with thinner inserts, further reflecting the impact of increased insert thickness. The large discrepancies during the initial stage (5–15 s), in particular, can be attributed to experimental thermal lag caused by heat accumulation in the thicker insert, an effect not captured by the simulation’s assumption of instantaneous heat transfer.

The overall trend in both methods showed a steady temperature rise from 5 s to approximately 20 s, followed by stabilization. However, the slope of the experimental curve was consistently lower than that of the simulation, indicating a slower actual heating rate. This is likely due to unmodeled heat dissipation effects, which are exacerbated in a configuration with a thick insert and deep cooling layer. In such cases, the higher overall thermal resistance leads to less uniform heat dispersion and a slower thermal response.

#### 3.1.5. Case 22: Insert Thickness: 2.5 mm—Cooling Layer Thickness: 2 mm—Water Flow Rate: 2.5 L/min—Coolant Temperature: 80 °C

**Figure 15:**
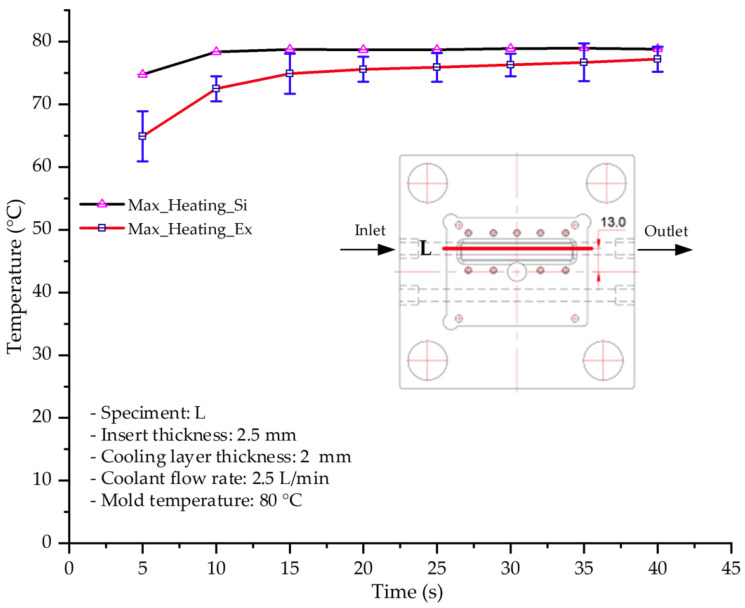
Comparison of maximum heating temperature between simulation and experiment for Case 22.

For the conditions of Case 22, a comparison between simulation and experiment indicates a generally consistent trend in temperature variation, albeit with notable discrepancies during the initial heating stage (Figure 15, Table 13 and Table 14). Specifically, the simulated maximum temperature rose from 74.76 °C at 5 s to 78.96 °C at 20 s, whereas the experimental measurement increased from 64.9 °C to 75.6 °C. Consequently, the percentage error decreased markedly from 15.19% at 5 s to 4.09% at 20 s, indicating that the deviation was primarily concentrated in the early heating phase, after which the two curves converged.

For the minimum temperature, the simulation yielded values between 55–70.12 °C, while the experimental results were slightly higher, ranging from 62–73.1 °C. The relative error similarly declined from 11.29% at 5 s to 4.08% at 20 s, reflecting differences in the initial heat transfer rate due to the complexities of the experimental setup. Notably, the simulated temperature curve consistently over-predicted the experimental results, suggesting that the numerical model tends to overestimate heat transfer efficiency. This effect was particularly pronounced with the thicker 2.5 mm insert, which increases thermal resistance and reduces heat distribution uniformity in practice.

Spatial temperature distributions further substantiate this observation. The simulation produced more distinct high-temperature regions, whereas the experimental results displayed more diffuse thermal patterns, influenced by realistic coolant dynamics and environmental heat loss. The temporal evolution curves indicate that both methods reached a quasi-steady state after approximately 20 s; however, the equilibrium temperature in the experiment was 2–3 °C lower than that in the simulation. These findings highlight the significant impact of unmodeled external factors—such as ambient heat dissipation, measurement uncertainties, and non-uniform thermal contact—which were not fully captured by the simulation model.

Among the 80 °C cases, Case 5 (0.5 mm insert, 10 mm cooling layer, 3.5 L/min flow rate) emerged as the most effective configuration. This configuration yielded a steady-state (40 s) temperature differential within the cavity of only ~3.6 °C, with a simulation–experiment deviation of 2.4–7.2%. This superior performance is attributed to the combination of a thin insert with a thick cooling layer and a high flow rate, which collectively facilitated efficient through-wall heat transfer and minimized thermal gradients [50,51]. In contrast, Case 8 (thicker insert) resulted in a larger cavity ΔT and greater temporal fluctuations, while Case 22 (thickest insert, thinnest cooling layer) exhibited delayed thermal stabilization. The lower temperature cases (17 and 1) were dismissed as they did not meet the technological requirements for thin-wall molding. Therefore, Case 5 represents the optimal balance, providing a high mold temperature while maintaining stable and uniform thermal distribution. It is thus recommended as the most suitable configuration for high-quality injection molding of PA6 and PA6/30 wt.% GF composites, particularly when surface finish and geometric accuracy are prioritized [52].

Analysis of the five investigated cases (Table 15) reveals distinct variations in thermal distribution resulting from different parameter combinations. As expected, increasing the coolant temperature from 40 °C (Case 1) to 80 °C (Cases 5, 8, 22) elevates the mold temperature, which in turn enhances melt flowability and reduces weld line defects, a finding consistent with previous work [53,54]. However, optimality requires not only a high temperature but also thermal uniformity and high predictive fidelity.

To further investigate the source of these discrepancies, a quantitative sensitivity analysis was performed. This involved systematically varying key boundary conditions, including the convection heat transfer coefficient (h, ±15%), inlet water temperature (±2 °C), and the insert’s thermal conductivity (±10%). The analysis revealed that modifying h alone reduced the maximum error from 17.7% to 12.4%. Concurrently refining h and the coolant temperature further lowered the deviation to ~10.8%. When all three parameters were adjusted, the simulation–experiment discrepancy was ultimately reduced to 9.5%. These results substantiate that the primary source of deviation arises from idealized boundary assumptions and uncontrolled environmental heat losses, underscoring the critical importance of incorporating more realistic operating conditions into future simulation models.

### 3.2. Displacement Analysis

#### 3.2.1. Displacement Behavior of PA6 + 0 wt.% GF

**Figure 16:**
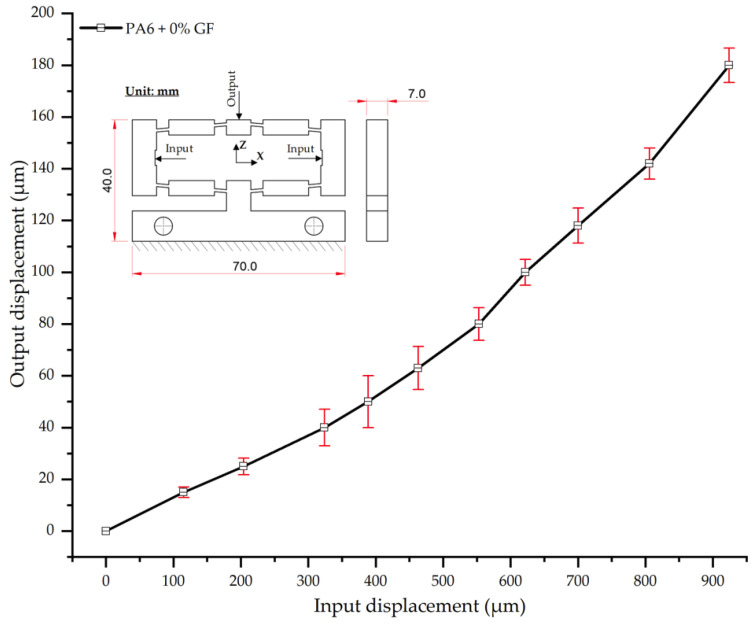
Input–output displacement relationship of PA6 without glass fiber (0 wt.% GF).

As shown in Table 16 and Figure 16, the experimental results for neat PA6 (0 wt.% GF) demonstrate a clear linear relationship between input and output displacement in the initial stage. This behavior is characteristic of the polymer matrix’s elastic response under an increasing applied load. Specifically, as the input displacement increased from 0 to 180 µm, the average output displacement increased proportionally from 0 to 924 µm.

The curve in Figure 16 indicates that while the trend is predominantly linear, the slope steepens at higher load levels (input displacement > 500 µm). This suggests the onset of nonlinear mechanical behavior, likely attributable to high stress and localized plastic deformation within the PA6 structure [52].

The small error bars (SD ranging from 2.0–10.1 µm) indicate acceptable repeatability among the measurements, confirming the reliability of the experimental results. The observed input–output displacement trend, which exhibits proportional amplification within the linear loading region, corroborates findings from previous studies on compliant mechanisms fabricated from pure polymer matrices, such as the 3D-printed bridge-type mechanism reported in [52]. However, in contrast with simulation-based findings in [55,56], our results show more pronounced nonlinear effects at higher load levels. This discrepancy is hypothesized to stem from residual stresses inherent to the injection molding process—a factor not typically considered in studies employing additive manufacturing or CNC machining. This finding highlights the significant influence of the manufacturing process on the mechanical response of PA6, particularly under high-load conditions.

#### 3.2.2. Displacement Behavior of PA6 + 5 wt.% GF

**Figure 17:**
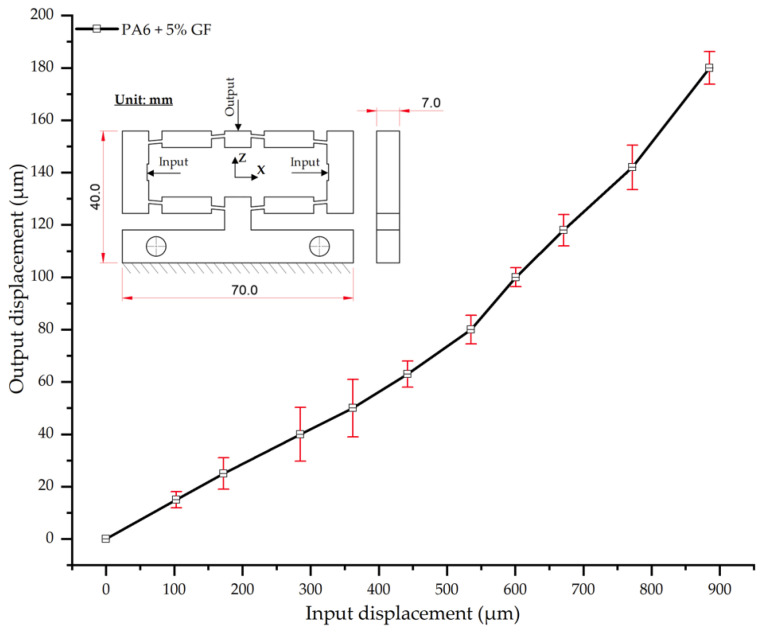
Input–output displacement relationship of PA6 + 5 wt.% GF.

As shown in Table 17 and Figure 17, the experimental results for PA6 reinforced with 5 wt.% glass fiber (PA6/5 wt.% GF) reveal that the input–output displacement relationship remains predominantly linear. However, the slope of the curve is notably reduced compared to that of neat PA6. Specifically, for an input displacement of 180 µm, the average output displacement was 885 µm—approximately 39 µm less than the 924 µm recorded for neat PA6. This reduction in displacement is attributed to the reinforcing effect of the glass fibers, which increases the composite’s elastic modulus and reduces its overall deformability.

The error bars (SD ranging from 3.1–11.0 µm) were slightly larger than those of the neat PA6 specimen, indicating increased data scatter. This is likely attributable to the non-uniform distribution of fibers within the polymer matrix. Nevertheless, these deviations remain within acceptable limits, affirming the reliability of the experimental results.

In agreement with previous studies [52,57], the observed reduction in displacement aligns with the fundamental reinforcement mechanism of composites, where glass fibers act as the primary load-bearing phase. As noted in [57,58], low fiber content (5–10%) can enhance stiffness without inducing excessive embrittlement, thus preserving a degree of elastic deformability. Compared with neat PA6, the PA6/5 wt.% GF specimen thus represents an effective trade-off, balancing improved mechanical stiffness with only a moderate reduction in deformation, all while maintaining stable linear behavior within the investigated loading range.

#### 3.2.3. Displacement Behavior of PA6 + 10 wt.% GF

**Figure 18:**
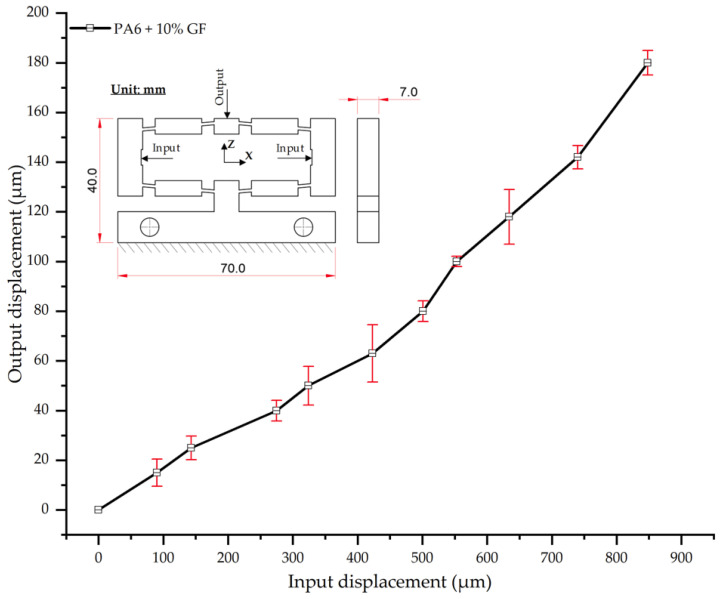
Input–output displacement relationship of PA6 + 10 wt.% GF.

As shown in Table 18 and Figure 18, the results for PA6 reinforced with 10 wt.% glass fiber (PA6/10 wt.% GF) reveal a persistent linear deformation trend. However, the overall displacement is further reduced compared to the composites with lower fiber content. At an input displacement of 180 µm, the average output displacement was 848 µm. This is 76 µm lower than that of neat PA6 (924 µm) and 37 µm lower than the PA6/5 wt.% GF composite (885 µm). This reduction in displacement clearly demonstrates that the increased glass fiber content enhances the material’s stiffness, restricts its elastic deformability, and reduces the input-to-output displacement amplification factor.

The experimental error bars (SD ranging from 2.1–11.5 µm) were comparable to the previous cases, indicating a relatively stable dataset. However, more pronounced deviations were observed at intermediate loading levels (e.g., 63–80 µm input), which may be attributable to either the non-uniform distribution of glass fibers or micro-scale interactions between the fibers and the polymer matrix.

These results corroborate the general trend wherein an increased glass fiber content enhances stiffness and elastic modulus, thereby reducing displacement [55,57]. For instance, ref. [57] reported an approximate 15–20% increase in the elastic modulus of PA6 with the addition of 10 wt.% GF. Furthermore, ref. [59] confirmed that the reinforcing effect is most pronounced in the 10–20% range, beyond which stress concentration effects may begin to degrade impact strength. Accordingly, the present findings confirm that the PA6/10 wt.% GF composite maintains a stable linear input–output relationship while reducing overall displacement relative to lower reinforcement levels. This affirms the suitability of this reinforcement ratio for applications demanding high stiffness and geometric stability.

#### 3.2.4. Displacement Behavior of PA6 + 15 wt.% GF

**Figure 19:**
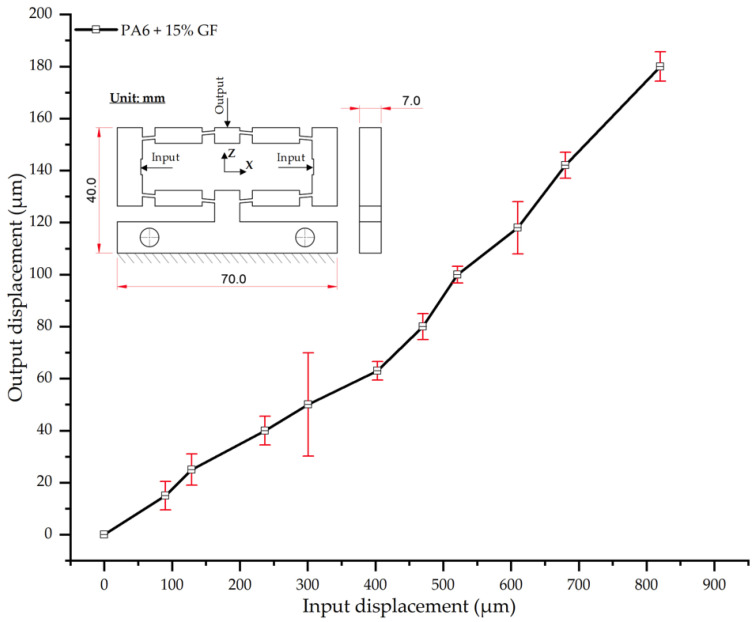
Input–output displacement relationship of PA6 + 15 wt.% GF.

The results for PA6 reinforced with 15 wt.% glass fiber (PA6/15 wt.% GF) further corroborate the trend of decreasing deformation with increasing fiber content (Table 19, Figure 19). At an input displacement of 180 µm, the average output displacement was 820 µm, a value lower than that of neat PA6 (924 µm), the PA6/5 wt.% GF composite (885 µm), and the PA6/10 wt.% GF composite (848 µm). This trend is a clear reflection of the reinforcing role of glass fibers, which enhance the composite’s stiffness and limit its elastic deformation.

Analysis of the displacement curve (Figure 19) shows that while the behavior is linear at very low loads, the experimental error increased significantly in the initial-to-intermediate loading region. For example, the standard deviation (SD) reached 19.9 µm at 50 µm of input and 19.3 µm at 100 µm of input. This increased variability is likely attributable to the non-uniform dispersion or local orientation of fibers within the matrix. However, at higher loads (>100 µm input), the error subsided to a stable range of 5.0–5.7 µm. This suggests a saturation effect in the stress transfer mechanism between the polymer matrix and the reinforcing fibers.

This trend corroborates the findings of [59], which reported that a GF content in the 15–20% range significantly enhances tensile stiffness and reduces the bending deformation of PA6. Similarly, ref. [57] noted that at 15 wt.% GF, PA6 achieves an optimal balance between mechanical reinforcement and stable deformation behavior. Nevertheless, as also observed in [60], the increased error at intermediate load levels likely stems from non-uniform fiber distribution. This underscores the importance of careful design and processing control for fiber-reinforced composites.

#### 3.2.5. Displacement Behavior of PA6 + 20 wt.% GF

**Figure 20:**
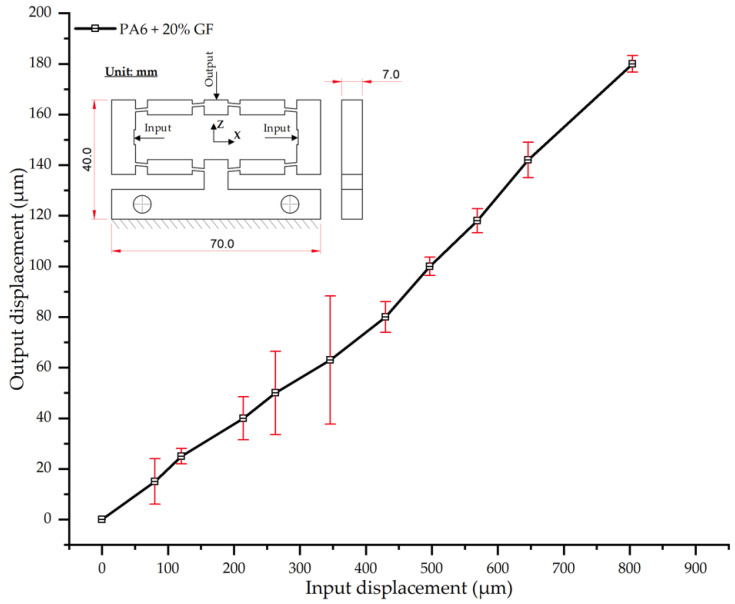
Input–output displacement relationship of PA6 + 20 wt.% GF.

The results for PA6 reinforced with 20 wt.% glass fiber (PA6/20 wt.% GF) further corroborate the trend of decreasing output displacement with increasing fiber content (Table 20, Figure 20). At an input displacement of 180 µm, the average output displacement was 804 µm, a value markedly lower than those of the composites with lower reinforcement levels (924 µm for neat PA6, down to 820 µm for PA6/15 wt.% GF). The steadily decreasing slope of the displacement curve reflects the enhanced stiffness and reduced elastic deformation imparted by the GF network. Regarding experimental repeatability, the standard deviation (SD) remained moderate (3–9 µm) across most load levels. However, a sharp increase in variability (SD ≈ 25.3 µm) was observed in the intermediate range (at 63 µm input). This fluctuation is hypothesized to correspond with a transition phase involving fiber reorientation and micro-mechanisms such as fiber–matrix debonding [57,60]. As the input displacement increased beyond 100 µm, the SD subsided to a stable range (3–7 µm), indicating the fiber network had achieved a stable load-bearing state.

Compared to the PA6/15 wt.% GF composite, the 20 wt.% GF sample yielded a further 2–3% reduction in output displacement at higher loads, confirming the trend of increasing stiffness. These results align with prior studies reporting that GF content up to ~20% significantly enhances the modulus of PA6, provided that microstructural uniformity and strong fiber–matrix adhesion are maintained [57,59]. Furthermore, the localized high SD values in the intermediate load region are consistent with observations in [60]. Such studies attribute these fluctuations to microstructural heterogeneities (e.g., fiber clustering, local adhesion variations) that can affect the mechanical response before a stable load-bearing state is reached.

#### 3.2.6. Displacement Behavior of PA6 + 25 wt.% GF

**Figure 21:**
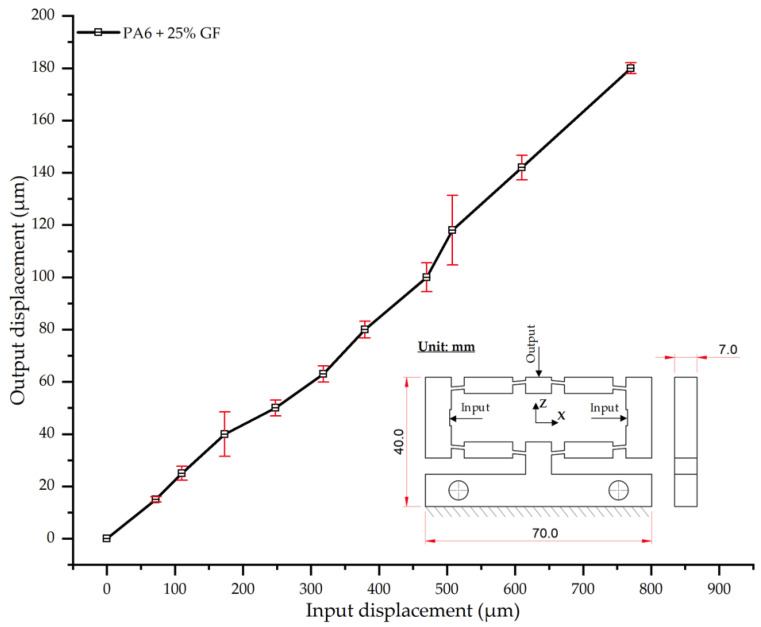
Input–output displacement relationship of PA6 + 25 wt.% GF.

As shown in Table 21 and Figure 21, the PA6 composite with 25 wt.% glass fiber (PA6/25 wt.% GF) demonstrates a significant reduction in output displacement compared to samples with lower fiber fractions. This confirms the strong mechanical reinforcement effect of the glass fibers. Specifically, at an input displacement of 180 µm, the average output displacement was 770 µm, markedly lower than that of neat PA6 (924 µm) and the 20 wt.% GF composite (804 µm). The displacement curve (Figure 21) maintains a near-linear trend, but its slope is shallower than that of composites with lower fiber content (<15 wt.% GF), reflecting a shift in load-bearing predominance from the polymer matrix to the glass fiber network.

In the low-load region (<200 µm), the standard deviation (SD) values remained within 2–8 µm, indicating high experimental stability. However, the SD peaked at 15.3 µm in the intermediate load region (~500 µm). This increased variability likely reflects localized non-uniformities in fiber dispersion or orientation, a phenomenon previously reported for PA6/GF composites [57,60]. Beyond this region, the SD decreased to a stable range of 2–5 µm, suggesting the material system had reached a more stable load-transfer state.

Compared with the 20 wt.% GF sample, the 25 wt.% GF composite yielded a further 4.2% reduction in output displacement at maximum load. However, it is crucial to note that such stiffness enhancement risks inducing embrittlement if the fiber fraction exceeds an optimal threshold [59]. The present results align with previous reports, which indicate that fiber contents in the 25–30 wt.% range often provide an optimal balance between stiffness enhancement and deformation reduction, while maintaining favorable processability [57,61].

#### 3.2.7. Displacement Behavior of PA6 + 30 wt.% GF

**Figure 22:**
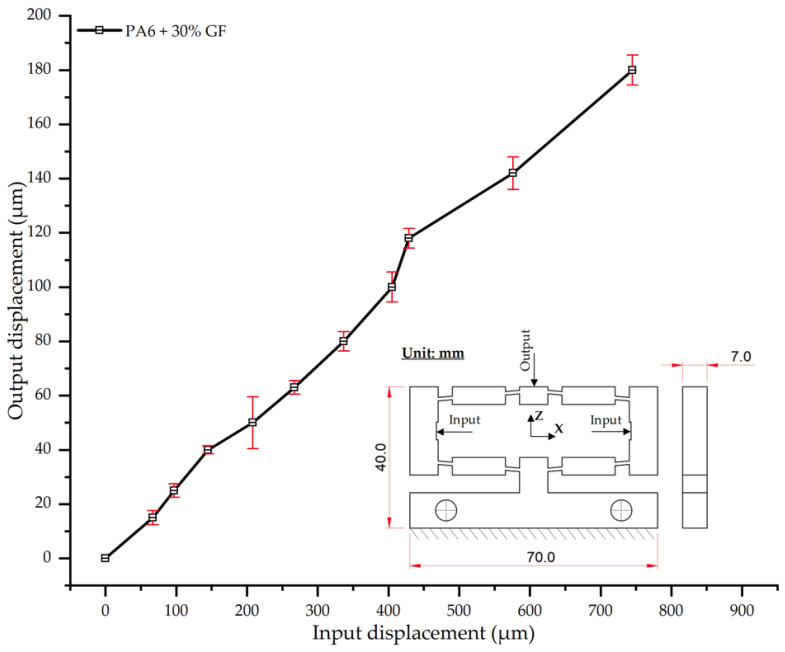
Input–output displacement relationship of PA6 + 30 wt.% GF.

The results for PA6 reinforced with 30 wt.% glass fiber (PA6/30 wt.% GF) demonstrate a pronounced reduction in output displacement compared to composites with lower fiber content, indicative of a substantial stiffening effect (Table 22, Figure 22). At an input displacement of 180 µm, the average output displacement was only 745 µm, markedly lower than that of neat PA6 (924 µm) and the 25 wt.% GF composite (770 µm). This result confirms that a 30% GF content significantly enhances the material’s stiffness and load-bearing capacity. The input–output curve (Figure 22) maintains a relatively linear trend, though its slope is considerably shallower than that of unreinforced PA6. Moreover, the standard deviation (SD) values remained low (2–6 µm), reflecting high system stability, with only a minor fluctuation (to 9.5 µm) in the intermediate range (300–500 µm), likely attributable to localized fiber dispersion non-uniformities [50,51].

Crucially, when compared with the 25 wt.% GF composite, the 30 wt.% GF sample showed only a marginal additional reduction of 3.2% in output displacement at maximum load. This suggests that the stiffening effect is approaching a saturation threshold. This finding corroborates previous studies, which have reported that reinforcement levels exceeding 30 wt.% GF often fail to yield proportional improvements in stiffness and may introduce risks of embrittlement and reduced impact toughness [57,62].

**Figure 23:**
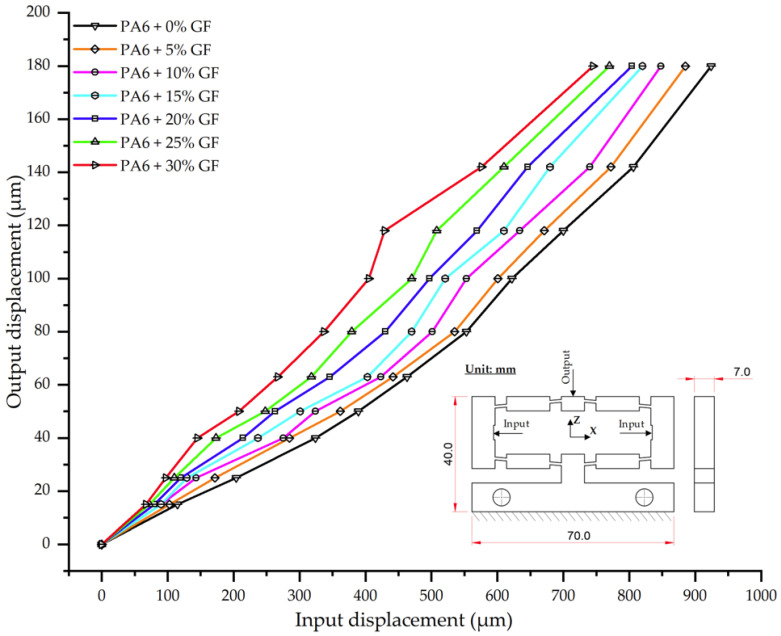
Combined input–output displacement curves for PA6 and PA6/GF composites.

As shown in Table 23 and Figure 23, glass fiber (GF) reinforcement exerts a significant influence on the input–output displacement of PA6 composites. While all displacement curves exhibit a linear trend, their slopes progressively decrease with increasing GF content, indicative of the composite’s stiffening effect. Specifically, at an input displacement of 180 µm, the average output displacement for neat PA6 was 924 µm. This value progressively decreased for the reinforced samples, with the PA6/30 wt.% GF composite recording the lowest displacement of 745 µm. These results indicate that 30 wt.% GF reinforcement reduced the material’s displacement by over 19% compared to the neat matrix, confirming the effectiveness of the fibers in enhancing stiffness and controlling deformation.

This trend corroborates previous findings [61,62,63,64], which show that while stiffness increases with fiber content, this effect is often accompanied by a risk of embrittlement above a ~25% threshold. This trade-off is reflected in the PA6/30 wt.% GF results, where the significant increase in stiffness likely compromises the material’s impact toughness. Another notable observation is that the divergence between the displacement curves becomes more pronounced at input loads above 500 µm. This indicates that the load-bearing mechanism of the GF network is activated more prominently at higher strain levels, where the fibers act as a structural skeleton constraining the polymer matrix.

Thus, the present results not only reinforce the established trends reported in the literature [42,50,51,65], but also provide systematic experimental evidence for PA6/PA6-GF composites under thin-walled injection molding conditions. It can be concluded that neat PA6 is suitable for applications requiring high ductility, PA6 + 15– 20 wt.% GF represents an optimal balance between stiffness and deformability, while PA6 + 30 wt.% GF is appropriate for engineering applications demanding high stiffness and dimensional stability, though caution must be exercised regarding potential embrittlement.

## 4. Conclusions

This study systematically investigated the effects of cooling layer integration and thin-walled mold design on the thermal and mechanical performance of PA6 and PA6/GF composites during thin-walled injection molding. By applying the Taguchi method with an L_25_ (5^4^) orthogonal array, the number of experimental trials was significantly reduced while maintaining statistical robustness. This facilitated the identification of the most influential parameters—insert thickness, cooling layer thickness, coolant flow rate, and coolant temperature—on mold temperature distribution and composite displacement behavior.

The results demonstrate that a thin insert (0.5 mm) combined with a 10 mm cooling layer and a high coolant flow rate (3.5 L/min) at 80 °C (Case 5) represents the optimal configuration. This setup not only elevates the mold surface temperature to improve melt flow and reduce weld-line defects, but also ensures a uniform temperature distribution with a maximum steady-state deviation of only 3.6 °C. Moreover, the deviation between simulation and experiment remained within 2.4–7.2%, affirming the predictive fidelity of the proposed model.

Thermo-mechanical analysis further revealed that the optimized cooling layer reduced residual stresses and warpage by up to 35% while simultaneously enhancing the dimensional stability of the composite samples. Increasing the glass fiber content from 0% to 30% progressively enhanced stiffness and reduced output displacement by nearly 19%, in agreement with established reinforcement mechanisms. However, a trade-off between stiffness and potential embrittlement was observed at high fiber ratios (>25%), highlighting the importance of balancing composite formulation with processing parameters.

The integration of a cooling layer with thin-walled inserts, optimized via a Taguchi-based design, provides a highly effective strategy for thermal management in thin-walled injection molding. This approach not only improves thermal uniformity and product quality but also yields practical insights into the processing of engineering composites like PA6/GF. These findings contribute to the development of advanced mold design strategies for next-generation injection molding applications where high precision, dimensional stability, and surface quality are paramount.

While this study demonstrates the efficacy of integrating a Cooling Layer system into a thin-walled insert for improving heat transfer, reducing ΔT, and controlling composite displacement, several limitations should be acknowledged. First, the number of experiments is constrained by the Taguchi design, which utilizes a small set of representative cases and thus does not cover the entire parameter space. Second, while the deviation between simulation and experiment is generally acceptable (2.4–7.2%), the results remain susceptible to practical factors such as ambient heat loss, material heterogeneity, and non-ideal thermal contact. Third, the current simulation model only considers steady-state heat transfer and does not yet incorporate detailed simulations of non-linear effects like turbulent flow or the residual stresses generated during rapid cooling.

Future work will aim to address these limitations. We plan to expand the experimental scope and perform a more rigorous statistical analysis via the Taguchi method, including ANOVA and Signal-to-Noise (S/N) ratio analysis. This will allow for a clearer quantification of each design factor’s influence on ΔT and displacement. Additionally, a fully coupled three-dimensional simulation model will be developed. This model will incorporate thermo-mechanical-flow interactions and the anisotropic behavior of the composite material. The ultimate goal is to enhance the model’s predictive capability and broaden the industrial applicability of the Cooling Layer system in high-precision, thin-walled injection molding.

## Figures and Tables

**Table 1:** Input experimental conditions for Taguchi design.

Level	Insert Thickness (mm)	Cooling Layer Thickness (mm)	Coolant Flow Rate (L/min)	Water Temperature (°C)
1	0.5	2	1.5	40
2	1.0	4	2.0	50
3	1.5	6	2.5	60
4	2.0	8	3.0	70
5	2.5	10	3.5	80

**Table 2:** Experimental conditions based on the Taguchi orthogonal array (L25).

No.	Insert Thickness (mm)	Cooling Layer Thickness (mm)	Coolant Flow Rate (L/min)	Water Temperature (°C)
1	0.5	2	1.5	40
2	0.5	4	2	50
3	0.5	6	2.5	60
4	0.5	8	3	70
5	0.5	10	3.5	80
6	1	4	1.5	60
7	1	6	2	70
8	1	8	2.5	80
9	1	10	3	40
10	1	2	3.5	50
11	1.5	6	1.5	80
12	1.5	8	2	40
13	1.5	10	2.5	50
14	1.5	2	3	60
15	1.5	4	3.5	70
16	2	8	1.5	50
17	2	10	2	60
18	2	2	2.5	70
19	2	4	3	80
20	2	6	3.5	40
21	2.5	10	1.5	70
22	2.5	2	2	80
23	2.5	4	2.5	40
24	2.5	6	3	50
25	2.5	8	3.5	60

**Table 3:** Material mixing ratios.

Case	Filler Ratio (wt.%)	PA6 /30 wt.%GF (g)	PA6 (g)	Total Weight (g)
1	0	0	1000	1000
2	5	166.7	833.3	1000
3	10	333.3	666.7	1000
4	15	500.0	500.0	1000
5	20	666.7	333.3	1000
6	25	833.3	166.7	1000
7	30	1000.0	0	1000

**Table 4:** Injection molding parameters.

Parameter	Unit	Simulation Input	Actual Experimental Value
Injection pressure	bar	39	1000
Packing pressure	bar	40	700
Filling time	s	2	2
Packing time	s	0.6	0.6
Cooling time	s	24	24
Melt temperature	°C	205	205
Mold temperature	°C	80	80
Injection speed	%	75	75

**Table 5:** Comparison of the temperature distribution inside the mold cavity between simulation and experiment for Case 1.

Time	Case 1	
5 s	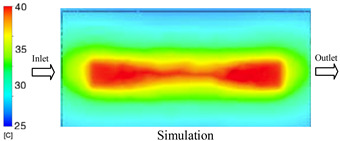	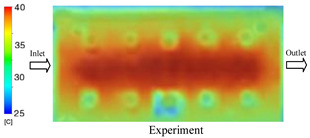
	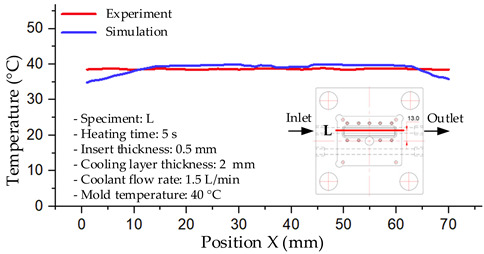	
20 s	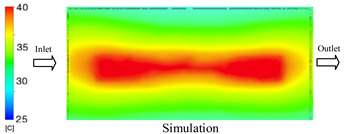	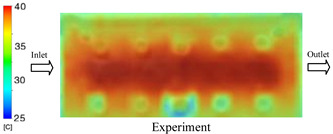
	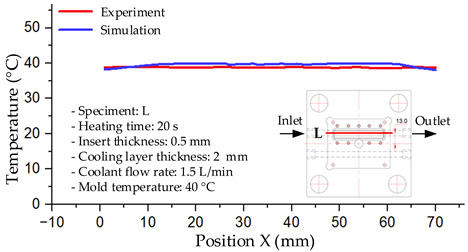	
40 s	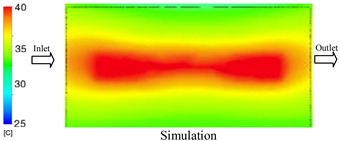	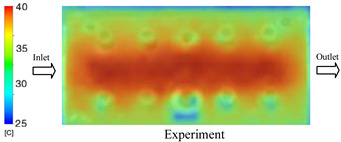
	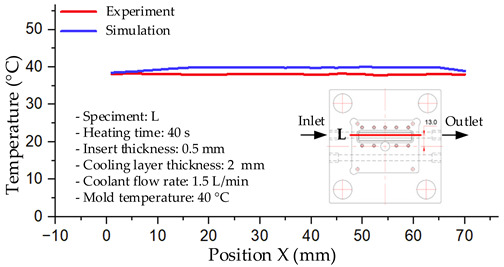	

**Table 6:** Comparison between simulation and experiment for case 1.

Time (s)	Simulation		Experiment		Error	
	Max Temperature (°C)	Min Temperature (°C)	Max Temperature (°C)	Min Temperature (°C)	Error_Max (%)	Error_Min (%)
5 s	39.99	34.84	38.9	38.4	2.80	9.27
10 s	39.99	37.3	39	38.5	2.54	3.12
15 s	39.98	37.86	39.2	38.4	1.99	1.41
20 s	39.98	38	39	38.6	2.51	1.55
25 s	39.98	38.22	39.1	38.4	2.25	0.47
30 s	39.98	38.33	39.3	37.7	1.73	1.67
35 s	39.99	38.47	39.4	37.6	1.50	2.31
40 s	40.08	38.52	39.3	37.8	1.98	1.90

**Table 7:** Comparison of the temperature distribution inside the mold cavity between simulation and experiment for Case 5.

Time	Case 5	
5 s	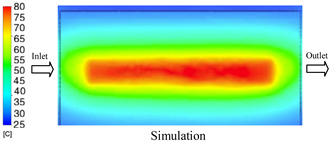	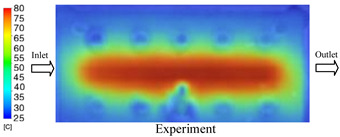
	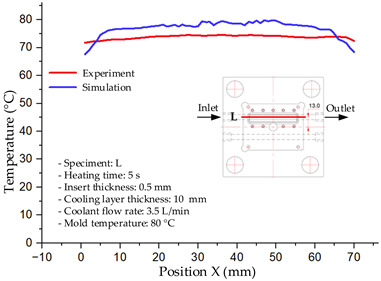	
20 s	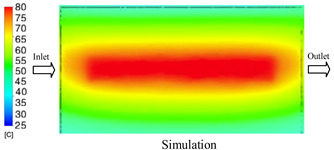	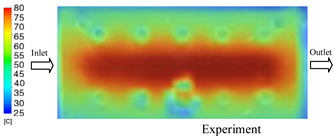
	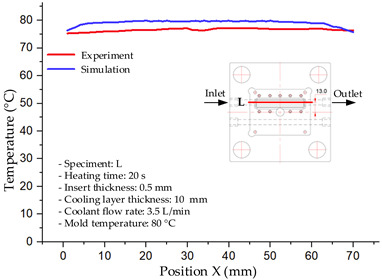	
40 s	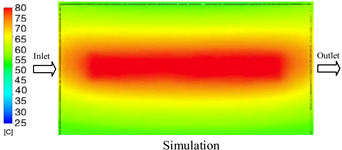	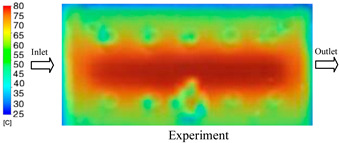
	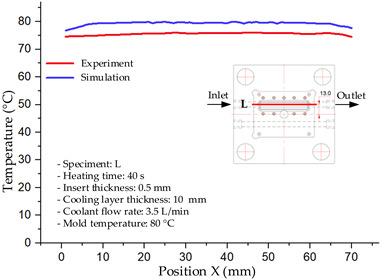	

**Table 8:** Comparison between simulation and experiment for case 5.

Time (s)	Simulation		Experiment		Error	
	Max Temperature (°C)	Min Temperature (°C)	Max Temperature (°C)	Min Temperature (°C)	Error_Max (%)	Error_Min (%)
5 s	79.86	67.6	74.5	71.8	7.19	5.85
10 s	79.91	75.1	75.9	71	5.28	5.77
15 s	80.4	75.23	77	74.1	4.42	1.52
20 s	79.93	77.76	77.2	75.3	3.54	3.27
25 s	79.94	76.42	76.8	74.9	4.09	2.03
30 s	79.94	76.52	77.9	74.4	2.62	2.85
35 s	79.95	76.55	77.7	74.1	2.90	3.31
40 s	79.94	76.78	78.1	74.5	2.36	3.06

**Table 9:** Comparison of the temperature distribution inside the mold cavity between simulation and experiment for Case 8.

Time	Case 8	
5 s	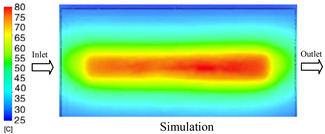	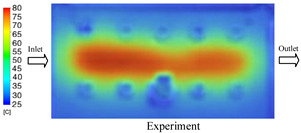
	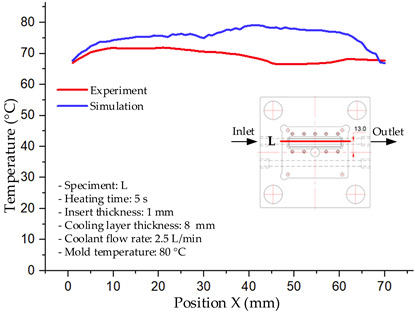	
20 s	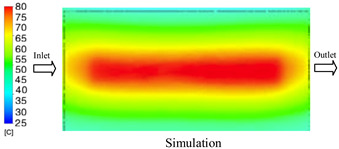	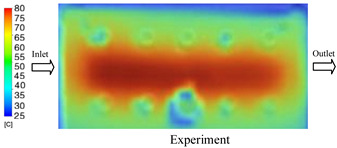
	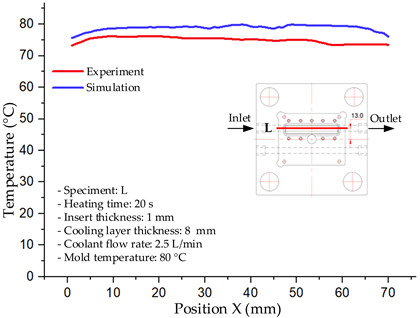	
40 s	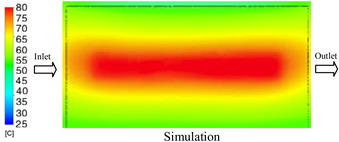	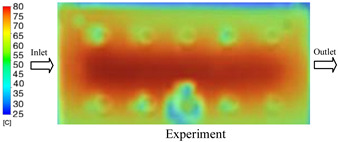
	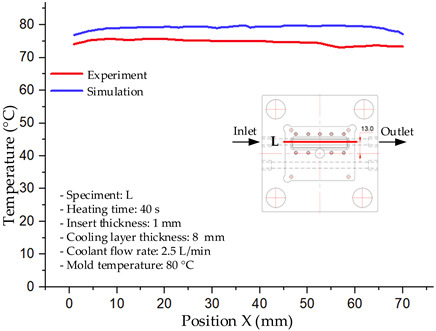	

**Table 10:** Comparison between simulation and experiment for case 8.

Time (s)	Simulation		Experiment		Error	
	Max Temperature (°C)	Min Temperature (°C)	Max Temperature (°C)	Min Temperature (°C)	Error_Max (%)	Error_Min (%)
5 s	79.13	66.88	71.9	66.6	10.06	0.42
10 s	79.94	74.71	74.6	70.9	7.16	5.37
15 s	79.8	75.4	75.3	72.1	5.98	4.58
20 s	79.9	75.7	76.2	73.3	4.86	3.27
25 s	79.82	76.36	76.8	74.1	3.93	3.05
30 s	79.81	76.44	76.6	74	4.19	3.30
35 s	79.85	76.9	76.4	73.7	4.52	4.34
40 s	79.88	76.95	76.7	73.1	4.15	5.27

**Table 11:** Comparison of the temperature distribution inside the mold cavity between simulation and experiment for Case 17.

Time	Case 17	
5 s	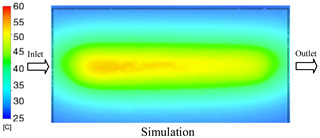	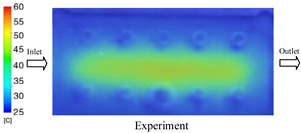
	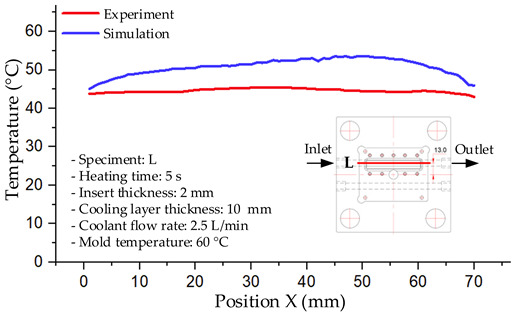	
20 s	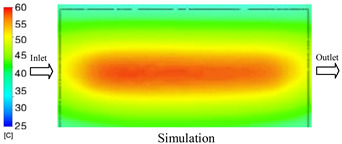	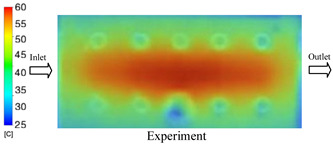
	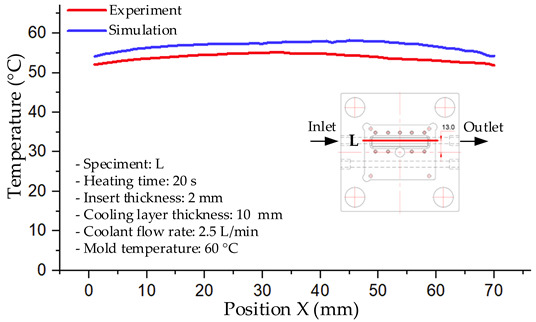	
40 s	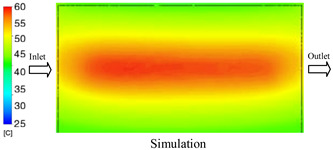	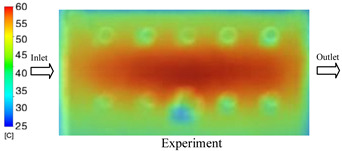
	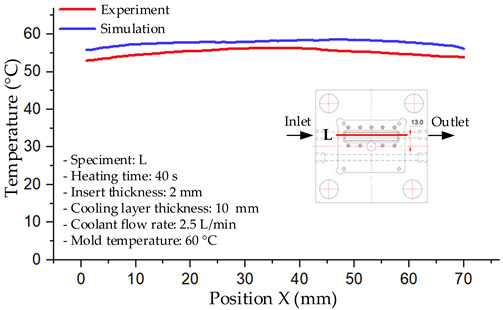	

**Table 12:** Comparison between simulation and experiment for case 17.

Time (s)	Simulation		Experiment		Error	
	Max Temperature (°C)	Min Temperature (°C)	Max Temperature (°C)	Min Temperature (°C)	Error_Max (%)	Error_Min (%)
5 s	53.57	45.06	45.5	43	17.74	4.79
10 s	58.41	51.94	52.6	46.2	11.05	12.42
15 s	58.28	53.6	55.1	51	5.77	5.10
20 s	58.22	54.12	55.2	51.9	5.47	4.28
25 s	58.33	54.45	55.3	52	5.48	4.71
30 s	58.46	54.96	55.3	51.9	5.71	5.90
35 s	58.6	55.79	55.9	51.9	4.83	7.50
40 s	58.69	55.83	56.4	53	4.06	5.34

**Table 13:** Comparison of the temperature distribution inside the mold cavity between simulation and experiment for Case 22.

Time	Case 22	
5 s	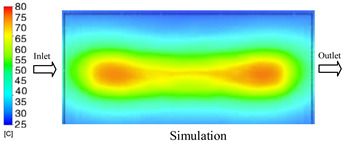	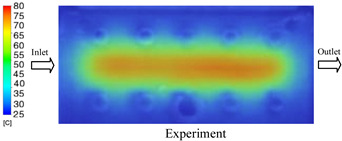
	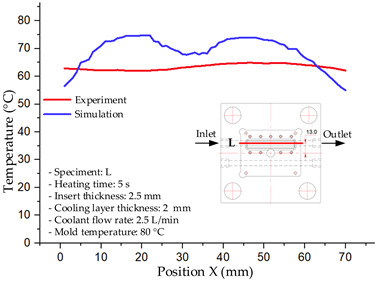	
20 s	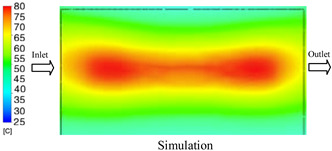	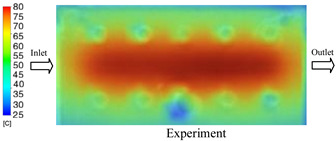
	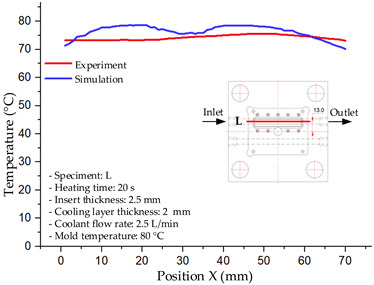	
40 s	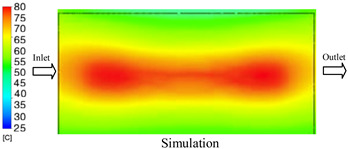	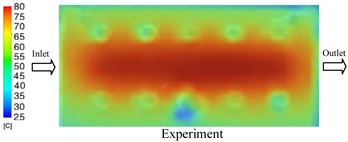
	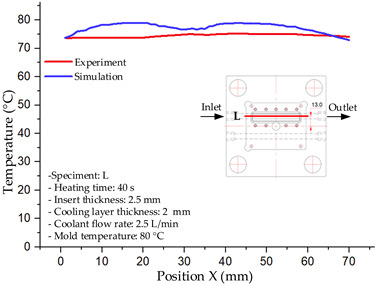	

**Table 14:** Comparison between simulation and experiment for case 22.

Time (s)	Simulation		Experiment		Error	
	Max Temperature (°C)	Min Temperature (°C)	Max Temperature (°C)	Min Temperature (°C)	Error_Max (%)	Error_Min (%)
5 s	74.76	55	64.9	62	15.19	11.29
10 s	78.35	66.21	72.5	67.9	8.07	2.49
15 s	78.74	68.96	74.9	71.6	5.13	3.69
20 s	78.69	70.12	75.6	73.1	4.09	4.08
25 s	78.7	70.95	75.9	73.4	3.69	3.34
30 s	78.89	72.13	76.3	74.1	3.39	2.66
35 s	78.96	72.7	76.7	74.1	2.95	1.89
40 s	78.83	74.02	77.2	73.6	2.11	0.57

**Table 15:** Comparison of five cases for thermal distribution analysis.

Case	Insert Thickness (mm)	Cooling Layer Thickness (mm)	Flow Rate (L/min)	Water Temperature (°C)	ΔT (Max–Min, Experiment)
1	0.5	2	1.5	40	1.5 °C (39.3/37.8)
5	0.5	10	3.5	80	3.6 °C (78.1/74.5)
8	1.0	8	2.5	80	3.6 °C (76.7/73.1)
17	2.0	10	2.5	60	3.4 °C (56.4/53.0)
22	2.5	2	2.0	80	3.6 °C (77.2/73.6)

**Table 16:** Input–output displacement measurements for PA6 without glass fiber (0 wt.% GF).

Input (µm)	0	15	25	40	50	63	80	100	118	142	180
Output 1 (µm)	0	115	203	331	388	456	558	627	692	805	922
Output 2 (µm)	0	117	208	317	380	472	555	617	702	812	931
Output 3 (µm)	0	113	202	323	400	460	546	622	705	800	918
Avg. Out (µm)	0	115	204	324	389	463	553	622	700	806	924
SD	0.0	2.0	3.2	7.0	10.1	8.3	6.2	5.0	6.8	6.0	6.7

**Table 17:** Input–output displacement measurements for PA6 with 5 wt.% GF.

Input (µm)	0	15	25	40	50	63	80	100	118	142	180
Output 1 (µm)	0	106	166	274	366	447	529	605	672	772	880
Output 2 (µm)	0	104	178	288	350	437	540	598	665	763	892
Output 3 (µm)	0	100	172	294	371	442	535	600	677	780	883
Avg. Out (µm)	0	103	172	285	362	442	535	601	671	772	885
SD	0.0	3.1	6.0	10.3	11.0	5.0	5.5	3.6	6.0	8.5	6.2

**Table 18:** Input–output displacement measurements for PA6 with 10 wt.% GF.

Input (µm)	0	15	25	40	50	63	80	100	118	142	180
Output 1 (µm)	0	90	138	270	318	423	506	551	645	742	850
Output 2 (µm)	0	85	147	276	322	435	498	554	635	744	842
Output 3 (µm)	0	96	145	278	333	412	500	555	623	735	851
Avg. Out (µm)	0	90	143	275	324	423	501	553	634	740	848
SD	0.0	5.5	4.7	4.2	7.8	11.5	4.2	2.1	11.0	4.7	4.9

**Table 19:** Input–output displacement measurements for PA6 with 15 wt.% GF.

Input (µm)	0	15	25	40	50	63	80	100	118	142	180
Output 1 (µm)	0	90	128	231	309	403	465	517	600	680	822
Output 2 (µm)	0	85	135	242	315	407	475	522	620	675	814
Output 3 (µm)	0	96	123	238	278	400	470	523	611	685	825
Avg. Out (µm)	0	90	129	237	301	403	470	521	610	680	820
SD	0.0	5.5	6.0	5.6	19.9	3.5	5.0	3.2	10.0	5.0	5.7

**Table 20:** Input–output displacement measurements for PA6 with 20 wt.% GF.

Input (µm)	0	15	25	40	50	63	80	100	118	142	180
Output 1 (µm)	0	70	117	215	268	337	431	493	571	639	800
Output 2 (µm)	0	85	121	205	277	375	435	498	573	645	805
Output 3 (µm)	0	86	123	222	245	327	423	500	564	653	806
Avg. Out (µm)	0	80	120	214	263	346	430	497	569	646	804
SD	0.0	9.0	3.1	8.5	16.5	25.3	6.1	3.6	4.7	7.0	3.2

**Table 21:** Input–output displacement measurements for PA6 with 25 wt.% GF.

Input (µm)	0	15	25	40	50	63	80	100	118	142	180
Output 1 (µm)	0	73	112	165	248	315	383	470	505	608	771
Output 2 (µm)	0	71	107	172	251	317	378	465	497	606	768
Output 3 (µm)	1	72	111	182	245	321	377	476	523	615	772
Avg. Out (µm)	0	72	110	173	248	318	379	470	508	610	770
SD	0.6	1.0	2.6	8.5	3.0	3.1	3.2	5.5	13.3	4.7	2.1

**Table 22:** Input–output displacement measurements for PA6 with 30 wt.% GF.

Input (µm)	0	15	25	40	50	63	80	100	118	142	180
Output 1 (µm)	0	65	100	145	198	270	340	400	430	575	751
Output 2 (µm)	0	66	97	144	217	265	337	405	425	570	740
Output 3 (µm)	0	70	95	147	209	267	333	411	432	582	745
Avg. Out (µm)	0	67	97	145	208	267	337	405	429	576	745
SD	0.0	2.6	2.5	1.5	9.5	2.5	3.5	5.5	3.6	6.0	5.5

**Table 23:** Summary of input–output displacement results for PA6 and PA6/GF composites.

Input (µm) Avg. Out (µm)	0	15	25	40	50	63	80	100	118	142	180
PA6 + 0 wt.% GF	0	115	204	324	389	463	553	622	700	806	924
PA6 + 5 wt.% GF	0	103	172	285	362	442	535	601	671	772	885
PA6 + 10 wt.% GF	0	90	143	275	324	423	501	553	634	740	848
PA6 + 15 wt.% GF	0	90	129	237	301	403	470	521	610	680	820
PA6 + 20 wt.% GF	0	80	120	214	263	346	430	497	569	646	804
PA6 + 25 wt.% GF	0	72	110	173	248	318	379	470	508	610	770
PA6 + 30 wt.% GF	0	67	97	145	208	267	337	405	429	576	745

## Data Availability

The original contributions presented in this study are included in the article. Further inquiries can be directed to the corresponding author.
